# Moment‐Equivalent Adhesion Statistics Inferred From Particle Resuspension Kinetics

**DOI:** 10.1002/smsc.70361

**Published:** 2026-08-11

**Authors:** Roy Almog, Ziv Klausner, Ronen Berkovich

**Affiliations:** ^1^ Department of Chemical Engineering Ben‐Gurion University of the Negev Beer‐Sheva Israel; ^2^ Department of Applied Mathematics Israel Institute for Biological Research Ness‐Ziona Israel; ^3^ Ilze Katz Institute for Nanoscience and Technology Ben‐Gurion University of the Negev Beer‐Sheva Israel

**Keywords:** adhesion forces, aerodynamic forces, resuspension, Rock‘n’Roll model

## Abstract

Particle resuspension from surfaces is a fundamental process in many chemical engineering systems, including aerosol dispersion, powder processing, filtration, contamination control, and environmental dispersion. Accurate prediction of resuspension requires knowledge of particle‐surface adhesion statistics, yet direct adhesion measurements are often technically demanding and strongly system‐dependent. Here, a data‐driven framework based on an augmented Rock‘n’Roll (RnR) model is developed to reconstruct adhesion‐force statistics from resuspension measurements. The present approach integrates a flexible bounded beta distribution into the RnR framework, allowing adhesion heterogeneity to be reconstructed without restricting the distribution to a lognormal form. It is shown here that this enables a robust, percentile‐resolved reconstruction of adhesion statistics that remain mechanically consistent with rolling detachment physics and aerodynamic forcing. Implementing this approach across multiple datasets and particle systems, the reconstructed adhesion percentiles were found to exhibit a systematic aerodynamic scaling when normalized by the rolling lever‐arm ratio. This reflects the linear contribution of rolling in the moment balance and serves as an internal consistency check of the inverse reconstruction. These results indicate that resuspension percentiles encode quantitative information about particle‐surface adhesion interactions. By isolating the mechanically relevant resistance to rolling detachment, the proposed method infers adhesion statistics without direct adhesion experiments.

## Graphical abstract

A data‐driven inverse framework is introduced to infer adhesion‐force statistics directly from particle resuspension measurements. By coupling the Rock’n’Roll model with a beta‐distribution formulation, adhesion percentiles collapse when normalized by rolling geometry. The results reveal predominantly drag‐controlled detachment and demonstrate that resuspension experiments can serve as an indirect probe of adhesion statistics.

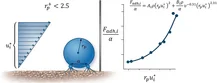

## 1 Introduction

The fate of particles residing on a surface is not necessarily permanent. Under suitable flow conditions, deposited particles can detach, re‐entrain, and return to the surrounding flow through a process known as resuspension. This phenomenon may influence natural and engineered environments, and is associated with environmental systems, industrial operations, and health‐related risks. Particle resuspension from surfaces is a fundamental phenomenon in many chemical engineering systems, including aerosol transport [1], powder processing [2, 3], filtration [4, 5], catalyst powder entrainment [6], and contamination control [7, 8]. The ability to predict and control such detachment processes is essential for understanding particulate transport, optimizing industrial operations, and mitigating environmental and health risks. Despite its broad relevance, quantitative prediction of resuspension remains challenging because particle removal is governed by the interplay between fluid forcing, particle mechanics, and heterogeneous particle‐surface adhesion interactions.

At its core, resuspension is initiated when aerodynamic forces overcome the resisting moments generated by particle‐surface adhesion. The onset of detachment therefore represents a critical threshold separating immobilized from mobile particulate matter [9, 10, 11, 12]. Numerous models have been proposed to describe this transition, accounting for surface roughness [13, 14], particle aggregation [15], transient flow effects [16, 17], and particle geometry including size, shape, and orientation [18, 19, 20, 21, 22, 23, 24, 25]. Among these factors, adhesion plays a central role [13, 26, 27] and is frequently estimated through contact mechanics models such as Johnson‐Kendall‐Roberts (JKR) [28] or Derjaguin‐Muller‐Toporov (DMT) [29].

However, adhesion between a particle and a rough surface cannot be represented by a single deterministic value. Surface heterogeneity, asperity geometry, and local contact mechanics generate a distribution of adhesion forces rather than a unique strength. Accurately characterizing this distribution is essential, as it directly determines the fraction of particles that detach under a given flow condition.

The RnR kinetic resuspension model [30], remains one of the most widely used frameworks for predicting resuspension. In its standard formulation, the adhesion force distribution is assumed to be lognormal, with its mean value derived from JKR theory and typically reduced to account for surface roughness. Although the lognormal distribution form is convenient, experimental studies demonstrate that particle‐surface adhesion can exhibit a variety of statistical shapes better represented by Gaussian, uniform, Weibull, or other parametric distributions [31, 32, 33]. Moreover, empirical adhesion distributions have been successfully incorporated into the RnR framework without strict reliance on contact mechanics assumptions for practical evaluation of the fraction of resuspended particles [34]. Recently, the beta distribution was shown to provide a generalized framework for adhesion which is bounded and flexible in its shape. Thus it is capable of accurately representing a wide range of experimentally measured adhesion force distributions [35].

In addition to adhesion statistics, resuspension models rely critically on a geometric parameter: the lever‐arm length, *a*, that defines the moment balance between aerodynamic and adhesive forces [13, 14, 18, 23, 36, 37, 38, 39]. Within the RnR formulation, the ratio between particle radius and lever arm, *r*_*p*_/*a*, and here denoted by *α*, strongly controls detachment kinetics. Despite its demonstrated sensitivity and statistical variability [40], *α* is often assumed to be a constant value of 100 across systems, regardless of system characteristics, following the evaluation of Reeks and Hall [24, 26, 30, 40, 41, 42, 43]. This widespread simplification introduces structural uncertainty, as *α* encapsulates complex surface morphology and contact geometry that vary between experiments.

Two fundamental constraints limit the predictive capability of current resuspension models: first is the reliance on a prescribed functional form for the adhesion force distribution, and second is the common assumption of a fixed geometric lever‐arm parameter. Both simplifications introduce structural uncertainty into the modeling framework. In this work, these limitations are overcome by developing a generalized, data‐driven reformulation of the RnR model that relaxes these assumptions and allows the governing parameters to be inferred directly from resuspension measurements.

First, instead of assuming the adhesion‐distribution shape, the beta distribution is used as a flexible bounded model, enabling asymmetric adhesion statistics to be represented without relying on a specific contact‐mechanics model. Adhesion distributions are reconstructed directly from experimental resuspension data using the inverse‐by‐optimization (IbO) algorithm [44], applied to nine independent resuspension datasets [20, 42, 44, 45, 46]. Unlike approaches that prescribe adhesion statistics a priori, the IbO framework infers effective adhesion distributions directly from measured resuspension behavior.

Second, the detachment criterion in terms of a normalized rolling coordinate is formulated: the ratio between adhesion force and the lever‐arm ratio, expressed as a moment‐equivalent mechanical parameter. In this representation, the geometric lever‐arm *a* is naturally embedded within the normalization rather than imposed as a fixed constant. This enables friction velocity to be directly correlated with adhesion‐force percentiles of resuspended particles, without requiring explicit numerical solution of the full RnR model for each case.

Building on the IbO derived adhesion statistics and the beta‐distributed framework, a generalized semi‐empirical formulation is developed, linking friction velocity to adhesion percentiles across a broad admissible range of *α* (10–1000), consistent with the variability reported by Brambilla and Brown [40]. The resulting framework does not prescribe a fixed adhesion law or geometric constant. Instead, it provides a transferable mapping between measurable flow conditions and the effective rolling‐resistance statistics that govern detachment.

In RnR‐style rolling, the relevant detachment competition is between aerodynamic rolling moment and adhesive resisting moment. The approach proposed here reduces this to a normalized adhesion ratio *F*_*α*_ = *F*_adh_/*α*, expressed as a mechanical coordinate for rolling (moment‐equivalent force per lever‐arm factor) containing *α*. By decoupling resuspension predictions from assumed adhesion distributions and fixed geometric parameters, this approach advances toward a data‐driven and experimentally anchored description of particle resuspension, suitable for cross‐study comparison and improved physical interpretability.

## 2 Theoretical Framework

### 2.1 Beta Distribution as a Unifying Statistical Framework

As a first step in refining the RnR model, the general beta distribution is incorporated. This modification provides a flexible framework for capturing the variability in particle‐surface adhesion across different systems. Owing to its bounded support and tunable shape parameters, the beta distribution can represent a wide range of adhesion force distributions, from narrow to highly skewed forms [35]. The probability density function (PDF) of the beta distribution for the random variable *x* ∈ [0, 1] is defined by [47]

(1) $$f\left(x;p,q\right)=\;\frac{1\;}{B(p,q)}x^{p-1}{\left(1-x\right)}^{q-1}$$

where the beta function $B(p,q)=\int_{0}^{1}t^{p-1}{\left(1-t\right)}^{q-1}\mathrm{dt}$ serves as a normalization constant. The two shape parameters of the beta distribution, *p* > 0 and *q* > 0, dictate the shape of the cumulative distribution function (CDF). For example, a unimodal distribution is characterized by both *p* > 1 and *q* > 1, reverse J‐shaped with *p* < 1 and *q* > 1, while a U‐shaped distribution is characterized by both *p* < 1 and *q* < 1 [47].

The parameters *p* and *q* are statistical shape parameters and should not be interpreted as direct physical descriptors of the particle–surface interaction. In the normalized adhesion coordinate, *p*/(*p *+ *q*) sets the mean location, *p* + *q* affects the breadth of the distribution, and the imbalance between *p* and *q* controls skewness. However, the dimensional adhesion‐force distribution also depends on *F*_min_ and *F*_max_, and different parameter sets can yield very similar CDFs. Therefore, the physically relevant reconstructed quantities in this work are the adhesion‐force CDF and its percentiles, rather than the individual beta parameters.

As standard beta distribution is defined within the finite interval [0, 1], it is possible to apply the beta distribution to a different finite range by using a linear transformation. For adhesion measurements, the transformation is defined by

(2) $$F_{\mathrm{adh}}=F_{\min}+x(F_{\max}-F_{\min})$$

where *F*_adh_ is a random variable describing the adhesion force, and *F*_min_ and *F*_max_ are the values of the minimal and maximal adhesion force, respectively. Therefore, each adhesion distribution is represented by its four characteristic parameters: *p*, *q*, *F*_min_, *F*_max_

(3) $$f\left(F_{\mathrm{adh}};p,q\right)=\;\frac{1\;}{\left(F_{\max}-F_{\min})B(p,q\right)}{\left(\frac{F_{\mathrm{adh}}-F_{\min}}{F_{\max}-F_{\min}}\right)}^{p-1}{\left(\frac{F_{\max}-F_{\mathrm{adh}}}{F_{\max}-F_{\min}}\right)}^{q-1}$$

Accordingly, the mean adhesion force and its variance are determined by the distribution's parameters

(4) $$\begin{array}{c} \left\langle F_{\mathrm{adh}}\right\rangle =F_{\min}+\left(F_{\max}-F_{\min}\right)\frac{p}{p+q}\\ \mathrm{Var}\left(F_{\mathrm{adh}}\right)={\left(F_{\max}-F_{\min}\right)}^{2}\frac{pq}{{\left(p+q\right)}^{2}\left(p+q+1\right)} \end{array}$$

While *F*_min_ and *F*_max_ define the physical boundaries, *p* and *q* account for the statistical heterogeneity. For this reason, the beta distribution naturally integrates with the resuspension modeling and the IbO framework, that will be described in the next section.

### 2.2 Rock’n Roll Model

The underlying beta adhesion distribution is evaluated from the measured resuspension data using the RnR model coupled with the IbO approach [30, 44]. The main components of this framework are summarized in the following.

In the RnR model, the remaining fraction of particles (Fr) is given by

(5) $$\mathrm{Fr}=\int_{0}^{\infty }\phi \left(F_{\mathrm{adh}}\right)e^{-k\tau }dF_{\mathrm{adh}}$$

where *ϕ*(*F*_adh_) is the distributed adhesion force probability density function (given by Equation (3)), *k* is the resuspension rate constant that quantifies the probability of a particle to be resuspended per time‐unit, and *τ* is the particles’ exposure time, that is, the period of time during which the particles are influenced by a given fluid velocity. Unless otherwise specified, *τ* = 1 s in the current analysis. This value was used in other studies, since the most particles are expected to detach in the first few milliseconds [14, 26, 42, 44].

The value of the resuspension rate constant in Equation (5) is determined by [30]

(6) $$k\left(F_{\mathrm{adh}}\right)=\;\left\{ \begin{array}{cc} n_{\theta } & for\;\frac{\left(F_{\mathrm{adh}}\;-\;\left\langle F\right\rangle \right)}{{\left\langle f^{2}\right\rangle }^{1/2}}\leq 0.75\\ \frac{n_{\theta }\exp\;\left[-\frac{{\left(F_{\mathrm{adh}}\;-\;\left\langle F\right\rangle \right)}^{2}}{2\left\langle f^{2}\right\rangle }\right]}{\frac{1}{2}\left[1+erf\left(\frac{F_{\mathrm{adh}}\;-\;\left\langle F\right\rangle }{\sqrt{2\left\langle f^{2}\right\rangle }}\right)\right]} & for\;\frac{\left(F_{\mathrm{adh}}\;-\;\left\langle F\right\rangle \right)}{{\left\langle f^{2}\right\rangle }^{1/2}}>0.75 \end{array}\right.$$

The fluctuating component $\sqrt{f^{2}}$ is usually taken as 0.2⟨*F*⟩ or measured [42], with the force couple

(7) $$\left\langle F\right\rangle =\alpha \left\langle F_{\text{drag}}\right\rangle +0.5\left\langle F_{\text{lift}}\right\rangle$$

being the aerodynamic force acting on the particle [30, 34]. This effective rolling force consists of standard rolling‐moment formulations in the RnR framework, where lift contributes to the rolling torque with reduced mechanical leverage.

The drag and lift forces are given by [30]

(8) $$\frac{\left\langle F_{\text{drag}}\right\rangle }{\rho \nu^{2}}=8{\left(\frac{2r_{p}u^{*}}{\nu }\right)}^{2}$$

(9) $$\frac{\left\langle F_{\text{lift}}\right\rangle }{\rho \nu^{2}}=4.2{\left(\frac{2r_{p}u^{*}}{\nu }\right)}^{2.31}$$

where *ρ* is the fluid's density, *ν* its kinematic viscosity, *r*_*p*_ is the particle radius, and *u* ^*^ is the friction velocity.

### 2.3 IbO Approach

The adhesion distribution is reconstructed directly from the resuspension dataset using the IbO algorithm, which enables a systematic exploration of the underlying parameter space [44]. For each specific set of input values that consists of the adhesion distribution as well as a value of *α* (= *r*_*p*_/*a*), the IbO algorithm generates a RnR‐based resuspension curve that describes the values of the flow friction velocities, *u* ^*^, that are required to resuspend given fractions of particles, *Rf* (= 1 − *Fr*). These model‐generated curves enable a direct comparison between the experimental *Rf*, or alternatively, the fractions remaining, *Fr*, and those predicted by the model at each friction velocity. For each specific *α*, there is an optimal parameter set of adhesion distribution that is defined as the one associated with the lowest Root Mean Square Error (RMSE) value, that is, the set that produces the minimal error between the experimental and model results.

Initially, the IbO algorithm was developed under the assumption that the underlying adhesion distribution follows the lognormal distribution, as originally adopted by Reeks and Hall [30]. However, the beta distribution was incorporated into the IbO framework as a generalized representation of the adhesion‐force distributions [35]. Within this setup, the beta distribution is introduced, having the following characteristic parameters: *F*_min_, *F*_max_, *p*, *q*, to the RnR model.

In this study, each run of the IbO algorithm was performed using a specified value of *α*. Rather than assuming *α* to be constant across the nine resuspension datasets, a range of *α* values was examined. The objective was to establish a generalized relation that allows any chosen *α* to be incorporated directly through a simple, closed‐form expression (see Sections 3.1 and 3.2 for more details).

Because inverse reconstruction may be nonunique, the fitted beta‐distribution parameters should not be interpreted as individually unique physical quantities. Instead, the relevant reconstructed object is the adhesion‐force CDF, from which adhesion percentiles are extracted. Practical identifiability was evaluated through a comparison of different beta‐parameter sets yielding similarly low reconstruction errors, demonstrated in the (Section S1). Although the parameter values may differ, the resulting CDFs and the corresponding RnR‐predicted resuspension curves nearly overlap. This indicates that the inverse problem is more robust at the level of adhesion percentiles than at the level of individual beta parameters. Therefore, the scaling analysis presented here is based only on percentile‐resolved adhesion forces, and not on the fitted beta parameters themselves.

### 2.4 Resuspension Experiments from Various Datasets

The IbO framework was evaluated using nine independent resuspension datasets reported in the literature [20, 42, 44, 45, 46]. These datasets were selected to satisfy the fundamental assumption of the RnR model, namely that particles are fully immersed within the viscous sublayer of the turbulent boundary‐layer flow. Under this constraint, the near‐wall velocity profile can be approximated as linear, and the aerodynamic torque acting on the particle is well described by the RnR formulation. Thus, the present analysis included experiments that satisfy this criterion. The selected datasets span a broad range of particle radii, particle‐surface material combinations, and flow conditions, thereby enabling assessment of scaling behavior across diverse systems that satisfy the underlying model assumptions.

The nine datasets used in the present analysis are summarized in Table 1. Here, each dataset corresponds to a specific particle‐size/material/substrate combination extracted from the literature and satisfying the viscous‐sublayer criterion required for the RnR formulation. The selected datasets cover particle radii from 4 to 36 µm, include glass, stainless‐steel, and barium‐titanate glass particles, and involve glass or silicon substrates.

**TABLE 1:** Summary of the experimental cases used in this study.

Dataset	Particle	*r*_*p*_ $\left[\mu m\right]$	Substrate
Taheri and Bragg [45]	Glass	10	Glass
Ibrahim et al. [20]	Soda‐lime glass	16	Glass
Ibrahim et al. [20]	Soda‐lime glass	36	Glass
Ibrahim et al. [20]	Stainless steel	35	Glass
Barth et al. [46]	Barium‐titanate glass	12.5	Glass
Barth et al. [46]	Barium‐titanate glass	22	Glass
Vincent et al. [42]	Soda‐lime glass	6	Silicon
Vincent et al. [42]	Soda‐lime glass	11.5	Silicon
Almog et al. [44]	Silica	4	Glass

*Note:* Each row corresponds to one particle‐size/material/substrate combination treated as an independent resuspension dataset in the present analysis.

Taheri and Bragg investigated resuspension of colloidal glass particles from glass substrates using turbulent flow generated by a rectangular nozzle [45]. Wall shear stress was measured using a Preston tube. Of the two particle sizes examined (10 and 17.5 µm), only the 10 µm particles satisfy the viscous sublayer requirement and are therefore retained in the present study.

Ibrahim et al. conducted wind‐tunnel resuspension experiments under accelerating airflow [20]. Their data for spherical soda‐lime glass particles (16 and 36 µm) and stainless steel particles (35 µm radius) deposited on glass slides were used. These datasets extend the size range and material diversity.

Barth et al. examined the influence of particle size and surface roughness in wind‐tunnel experiments [46]. Here, the results for barium‐titanate glass (GBM) particles of 12.5 and 22 µm radius deposited on smooth glass surfaces and exposed to constant turbulent flow for 40 s were used.

Vincent et al. measured progressive detachment of glass particles from silicon surfaces under increasing airflow [42]. Their data for particles of 11.5 and 6 µm radius were retained. Smaller particles were excluded because their resuspension fractions remained below 30%, failing to provide sufficient dynamic range for reliable adhesion reconstruction.

Finally, Almog et al. reported resuspension for spherical glass particles of 4 µm radius from glass surfaces in a wind‐duct facility [44]. These measurements are particularly relevant because adhesion distributions were previously reconstructed using the IbO approach and thus provide a benchmark for validation.

Together, these datasets (summarized in Table 1) provide a controlled yet diverse basis for investigating the interplay between particle size, aerodynamic forcing, adhesion heterogeneity, and the lever‐arm parameter α within the generalized RnR‐IbO framework.

## 3 Results and Discussion

### 3.1 Adhesion Inverse Reconstructions and *α*‐Dependence

**FIGURE 1:**
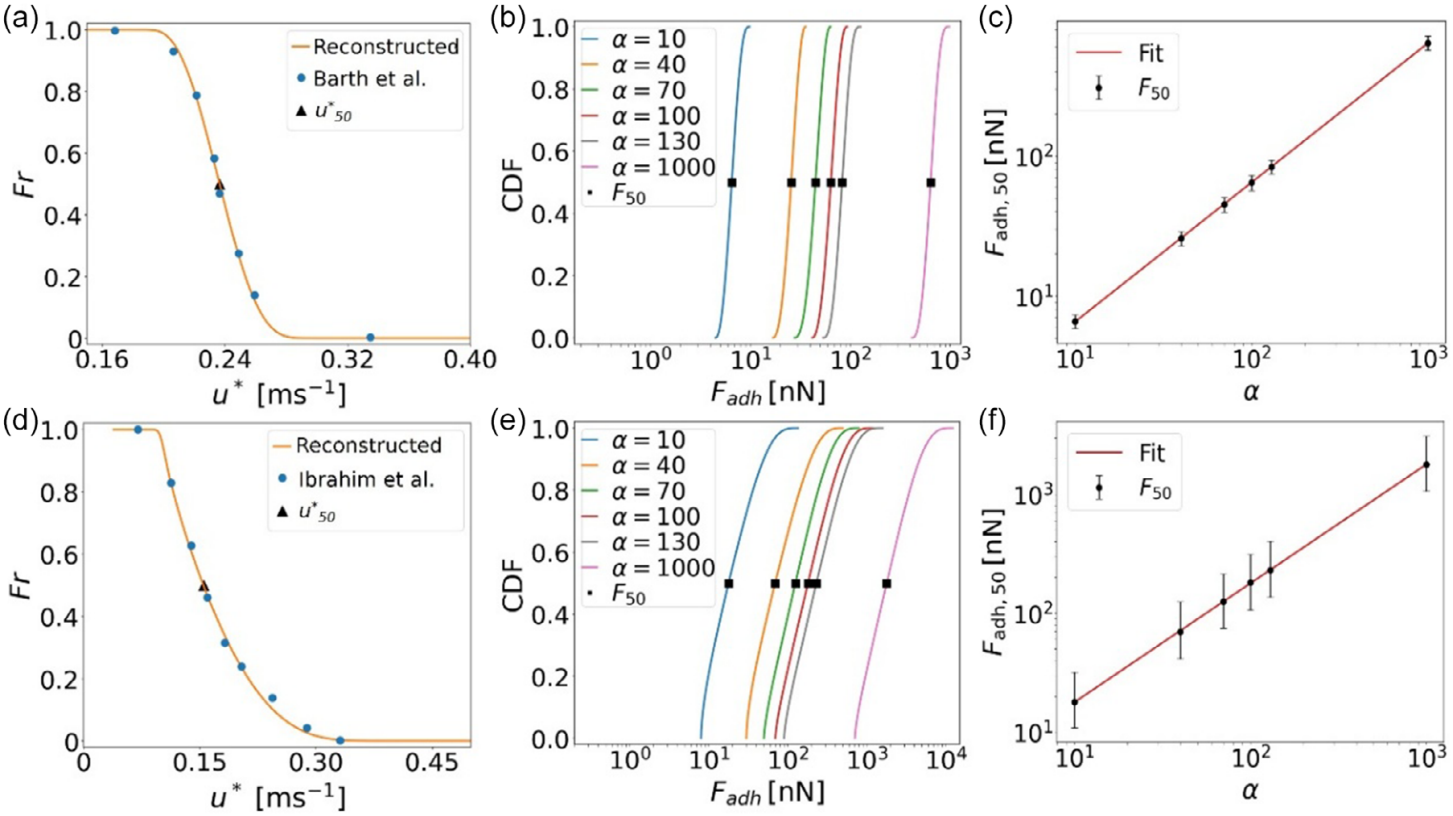
Representative inverse reconstructions and *α*‐dependence. (a) Measured fraction remaining Fr from Barth et al. [46] (12.5 µm GBM particles) and the corresponding RnR curve reconstructed using the IbO algorithm at *α* = 100. (b) IbO Reconstructed adhesion CDFs obtained for different prescribed values of *α*. Increasing *α* systematically shifts the inferred adhesion forces to higher values. Medians are marked by black square symbols at each CDF. (c) Median adhesion force, *F*_adh,50_ (black squares in (b)), as a function of *α*, showing an approximately linear scaling consistent with the rolling‐moment balance. (d) Measured fraction remaining Fr from Ibrahim et al. [20] (35 µm stainless‐steel particles) and corresponding IbO reconstruction at *α* = 100. (e) Reconstructed adhesion CDFs for varying *α*, demonstrating the same systematic shift observed in panel (b). Medians are marked by black square symbols at each CDF. (f) Linear dependence of *F*_adh,50_ on *α* for this dataset, confirming the generality of the compensatory scaling.

To illustrate the inverse reconstructions and their dependence on *α*, the workflow is first applied to two representative datasets—Barth et al. (12.5 µm GBM particles) in Figure 1a–c [46] and Ibrahim et al. (35 µm stainless‐steel particles) in Figure 1d–f [20]—and then the analysis is generalized across all nine datasets. Figure 1a compares the measured fraction remaining Fr from Barth et al. (blue circles) [46], with RnR curve reconstructed by the IbO algorithm for *α* = 100 (solid orange line) [30]. Agreement is maintained across the full friction‐velocity range, and the reference point Fr = 0.5 defines the corresponding friction velocity, *u*_50_^*^ (indicated by a black triangle symbol).

Re‐executing the IbO algorithm while prescribing different values of *α* yields a family of reconstructed adhesion cumulative distribution functions (CDFs) (Figure 1b) with their corresponding beta distributions’ parameters (Table 2). Despite variations in the beta shape parameters *p* and *q*, all extracted adhesion distributions retain a unimodal shape characterized by a positive skew (1 < *p* < *q*). No systematic trend is observed in the values of *p* and *q* themselves.

**TABLE 2:** Beta parameters for each extracted distribution (Barth et al. [46] 12.5 μm radius particles).

*α*	*F*_min_ $\left[nN\right]$	*F*_max_ $\left[nN\right]$	*p*	*q*
10	4.5	9.9	2.2	3.4
40	17	36	2.2	2.5
70	28	64	2.4	2.6
100	42	94	2.2	2.8
130	54	128	2.7	3.9
1000	419	990	2.7	4

Figure 1c shows the median adhesion force, *F*_adh,50_, extracted from the *α*‐dependent reconstructed CDFs in Figure 1b and plotted as a function of *α*. The associated uncertainty is represented by the interquartile range (IQR), defined as *F*_adh,75_ − *F*_adh,25_ for each reconstructed distribution. As *α* increases, the IQR broadens, indicating a widening of the adhesion‐force distributions, and reflected by increasing *F*_min_ and *F*_max_. In contrast, the median adhesion force exhibits a clear linear dependence on *α*, with *F*_adh,50_ = 6.4 × 10^−10^ *α* + 8.1 × 10^−11^, and *R* ^2^ = 1. A possible explanation for this trend can be the fact that *α* scales the aerodynamic contribution in the RnR rolling‐moment balance. Therefore, in order to achieve the same resuspension kinetics under a larger *α* and a constant resuspension rate, *k*, requires proportionally larger inferred adhesion forces (Equations (5)–(7)).

To verify that the observed linear *F*_adh,50_(*α*) scaling is not specific to one dataset or distribution shape, the same procedure was repeated for the stainless‐steel dataset of Ibrahim et al. [20] The corresponding fit, reconstructed adhesion CDFs, and resulting *F*_adh,50_ as a function of *α* are shown in Figure 1d–f (here with *F*_adh,50_ = 1.8 × 10^−9^ *α* − 7.1 × 10^−10^ and also *R* ^2^ = 1) and Table 3. The beta parameters are reported as one representative parametrization of the reconstructed CDFs and are not interpreted as individually unique physical observables.

**TABLE 3:** Beta parameters for each extracted distribution (Ibrahim et al. [20] 35 μm radius particles).

*α*	*F*_min_ $\left[nN\right]$	*F*_max_ $\left[nN\right]$	*p*	*q*
10	8	135	0.6	4.1
40	30	500	0.6	3.8
70	50	800	0.7	4.0
100	70	1200	0.7	4.1
130	90	1600	0.7	4.3
1000	710	12,300	0.7	4.3

Notably, the linear dependence persists (Figure 1f), despite a qualitatively different inferred beta distributions’ shape (reverse J‐shaped: *p* < 1, *q* > 1). This linear behavior demonstrates the robustness of the inverse algorithm and of the underlying linear scaling, particularly given that the second dataset is characterized by a different beta distribution's shape.

To evaluate the replacement of the conventional lognormal adhesion‐force distribution by the bounded beta distribution introduced in this work, a direct beta‐lognormal comparison is provided in the (Section S2). In this comparison, both distributions were optimized within the same RnR‐based inverse framework and assessed against the measured resuspension curves using RMSE. For the nine datasets analyzed, the beta formulation yielded lower RMSE values than the lognormal formulation in all cases. When averaged across all nine datasets, the mean RMSE decreased from approximately 0.0291 for the lognormal reconstructions to approximately 0.0173 for the beta reconstructions. These results support the use of the beta distribution as a flexible bounded reconstruction tool, without implying that the lognormal distribution is generally invalid. In cases where the experimental data does not constrain the full resuspension transition, differences between the beta and lognormal CDFs outside the measured range should be interpreted with caution.

The adhesion forces inferred from the inverse RnR analysis were then examined for consistency with independently measured adhesion forces reported for comparable particle‐surface systems. For the Ibrahim et al. [20] dataset with *r*_*p*_ = 16 μm, the reconstructed median adhesion forces for *r*_*p*_/*a* = 130, 100, and 70 were approximately 890, 680, and 480 nN, respectively. Using the estimate *r*_*p*_/*a* ≈ 90, expected for a smooth surface from Brambilla and Brown [40], gives a median adhesion force of approximately 610 nN, which lies within the range reported by Biggs et al. [48] for glass particles on glass surfaces. For the 4 μm‐radius glass‐colloid dataset from Almog et al. [44] the reconstructed median adhesion forces for *r*_*p*_/*a* = 100, 70, and 40 were approximately 1260, 890, and 510 nN, respectively. Using the corresponding estimated value *r*_*p*_/*a* ≈ 50 gives a median adhesion force of approximately 640 nN, which is comparable in magnitude to the adhesion forces reported by Rush et al. [31] for glass particles on glass surfaces.

A summary of the literature‐based consistency checks is provided in the (Section S3). The comparison is not intended as a direct validation of the absolute adhesion forces, because the reconstructed values depend on the assumed rolling lever arm and represent effective moment‐equivalent adhesion resistances. Nevertheless, the reconstructed median values for representative glass–glass systems are comparable in magnitude to independently reported adhesion measurements, supporting the physical plausibility of the inverse reconstruction.

The reconstructed adhesion forces should be interpreted as effective, moment‐equivalent adhesion resistances associated with rolling detachment rather than as direct pull‐off forces. This distinction is important because most independent adhesion measurements, such as AFM force spectroscopy or centrifugal detachment, probe different mechanical pathways and are often performed on different surface areas, roughness states, humidity conditions, and particle histories. Therefore, in the absence of paired resuspension and direct adhesion measurements for the same particle‐surface systems, validation of the absolute reconstructed adhesion forces can only be performed through approximate consistency checks. For representative glass–glass systems, the reconstructed median adhesion forces fall within the same order of magnitude as values reported from direct adhesion measurements in the literature. This agreement supports the physical plausibility of the inverse reconstruction. However, because the absolute values depend on the assumed lever‐arm ratio *r*_*p*_/*a*, the main robust outcome of the present analysis is the *α*‐normalized moment‐equivalent adhesion resistance and its scaling with friction velocity, rather than a unique direct measurement of the absolute pull‐off adhesion force.

### 3.2 Data‐Driven Extension of the RnR Model: *α*‐Collapse of Normalized Adhesion Percentiles

In the original RnR formulation, the geometric lever‐arm parameter *α* = *r*_*p*_/*a* is typically treated as a fixed quantity. However, as was demonstrated in the previous section, *α* directly scales the aerodynamic rolling moment, and therefore its assumed value influences the reconstructed adhesion distribution obtained via the IbO algorithm. To systematically assess this dependence, the examination of the treatment of the parameter *α* as a variable is further expanded.

For each of the nine resuspension datasets described in Section 2.5, six values of *α* ranging from 10 to 1000 were prescribed. For every (dataset, *α*) pair, the IbO algorithm reconstructed an effective adhesion force distribution that reproduces the measured resuspension curve. In total, 54 adhesion force CDFs (9 datasets × 6 values of *α*) were obtained.

After establishing the systematic *α*‐dependence of the reconstructed median adhesion forces in Section 3.1, percentile‐resolved adhesion forces, *F*_adh,*i*_ (*i* ∈ [5,95]), were extracted for each reconstructed CDF at each prescribed *α*, for all nine datasets. Each percentile corresponds, through the RnR model, to a specific friction velocity, *u*_i_^*^, and resuspended fraction *Rf*_*i*_. This pairing establishes a direct mapping between the adhesion force and the aerodynamic conditions required for detachment, which is analyzed using Pearson correlations.

To isolate the influence of *α*, the normalized adhesion force is defined as

(10) $$F_{\alpha ,i}\equiv \frac{F_{\mathrm{adh},i}}{\alpha }$$

This normalization reflects the fact that adhesion enters the rolling‐moment balance through the ratio *F*_adh_/*α*. If the reconstructed adhesion statistics are physically consistent, *F*_*α*,*i*_ should exhibit invariant scaling behavior across different values of *α*.

Motivated by the aerodynamic force expressions in Equations (7) – (9) that show that the aerodynamic force ⟨*F*⟩ is proportional to (*r*_*p*_ *u* ^*^)^*n*^ with *n* ≥ 2 and by the torque balance underlying rolling detachment, the scaling relationship between $F_{\alpha ,i}$ and $r_{p}u_{i}^{*}$ for the nine datasets is examined. Thus, Pearson correlation coefficient is used in order to assess the consistency of the following proportionality

(11) $$F_{\alpha ,i}\propto {\left(r_{p}u_{i}^{*}\right)}^{n_{i}}$$

This formulation enables a direct correlation‐based evaluation of whether the normalized reconstructed adhesion forces conform to the expected aerodynamic scaling, without imposing a priori constraints on the value of *α*.

A central implication of this analysis concerns the role of the geometric lever‐arm parameter *α*. If the inferred scaling exponent *n*_*i*_ (for *i* ∈ [5,95]) and the corresponding Pearson correlation strength, *r*, remain consistent across different prescribed values of *α*, this indicates that the reconstructed adhesion statistics reflect a physically meaningful torque balance, rather than compensating for an assumed geometry. In this sense, *α*‐invariance of the normalized scaling serves as a self‐consistency criterion for the model. Conversely, a strong dependence of *F*_*α*,*i*_ on *α* would imply that the predictive capability of the RnR framework is contingent on a priori geometric assumptions, thereby limiting its generalizability.

**FIGURE 2:**
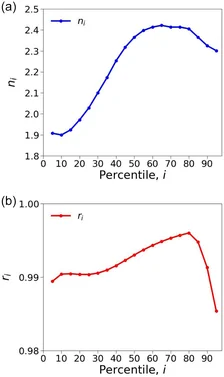
Percentile‐dependent aerodynamic scaling exponents and correlation strengths for normalized adhesion forces. (a) Extracted scaling exponent, *n*_*i*_, corresponding to the maximum Pearson correlation between *F*_*α*,*i*_ and ${\left(r_{p}{u^{*}}_{i}\right)}^{n_{i}}$ for each percentile, *i*. (b) Corresponding maximum Pearson correlation coefficient, *r*_*i*_, obtained for each percentile. The results indicate that the optimal scaling exponent lies between 1.9 and 2.4 over the examined percentile range.

Figure 2a presents the extracted scaling exponents *n*_*i*_ that yielded the highest correlation between *F* *_α_* *_,_* *_i_* and ${\left(r_{p}{u^{*}}_{i}\right)}^{n_{i}}$ as manifested by the corresponding highest *r*_*i*_ value for each percentile, shown in Figure 2b. Across all percentiles, the fitted exponent lies in the range 1.9  ≤ *n*_*i*_ ≤ 2.4, while the correlation strength remains consistently high (*r *> 0.985), indicating a robust and well‐defined scaling relationship. This behavior supports the presence of an intrinsic aerodynamic scaling, rather than a result imposed by the regression procedure.

The exponents range of 1.9 < *n*_*i*_ < 2.4 indicating an approximately quadratic scaling of normalized adhesion force with $r_{p}u^{*}$. This behavior is physically aligned with the drag and lift scaling in the viscous sublayer: from Equation (8), $\langle F_{drag}\rangle \propto \rho r_{p}^{2}{u^{*}}^{2}$, i.e., quadratic in $\left(r_{p}u^{*}\right)$, and the lift contribution (Equation (9)) introduces a slightly higher exponent of 2.31 and an additional dependence on *ν* ^−0.31^. However, its moment contribution is reduced in Equation (7) relative to the drag term, particularly for the range of *α* values considered here.

The similarity between the range of *n*_*i*_ values in Figure 2a and those associated with the drag and lift terms allows for relating the aerodynamic term (left‐hand side of Equation (7) ) to the normalized adhesion force (left‐hand side of Equation (11) ), thereby setting the basis for the regression presented in the following section.

### 3.3 Drag‐Lift Decomposition and Identifiability

After establishing the correlation between $F_{\alpha ,i}$ and $r_{p}u_{i}^{*}$ which exhibited its highest values for *n*_*i*_ values similar to those associated with the drag and lift contributions, as noted in Equations (8) and (9), the correlation analysis presented in the previous section is used to determine the regression parameters of the following form

(12) $$F_{\alpha ,i}=A_{i}\rho {\left(r_{p}u_{i}^{*}\right)}^{2}+\frac{B_{i}\rho }{\alpha }\upsilon^{-0.31}{\left(r_{p}u_{i}^{*}\right)}^{2.31}$$

In this formulation, *A*_*i*_ denotes the percentile‐resolved coefficient associated with drag‐controlled rolling resistance, while *B*_*i*_ captures the contribution of lift‐sensitive effects. This separation allows a direct physical interpretation of the detachment mechanism. When *B*_*i*_ ∼ 0, removal is governed predominantly by drag‐induced torque, consistent with a quasistatic rolling scenario and an effective scaling exponent close to *n* ∼ 2. If *B*_*i*_ becomes appreciable only at the lower percentiles, it suggests that the weakest‐adhering particles detach through lift‐assisted or fluctuation‐enhanced events, whereas the bulk of the population remains drag‐dominated. Conversely, if *B*_*i*_ remains systematically non‐negligible across percentiles, lift must be regarded as an intrinsic component of the detachment mechanism and therefore explicitly retained in predictive resuspension modeling.

**FIGURE 3:**
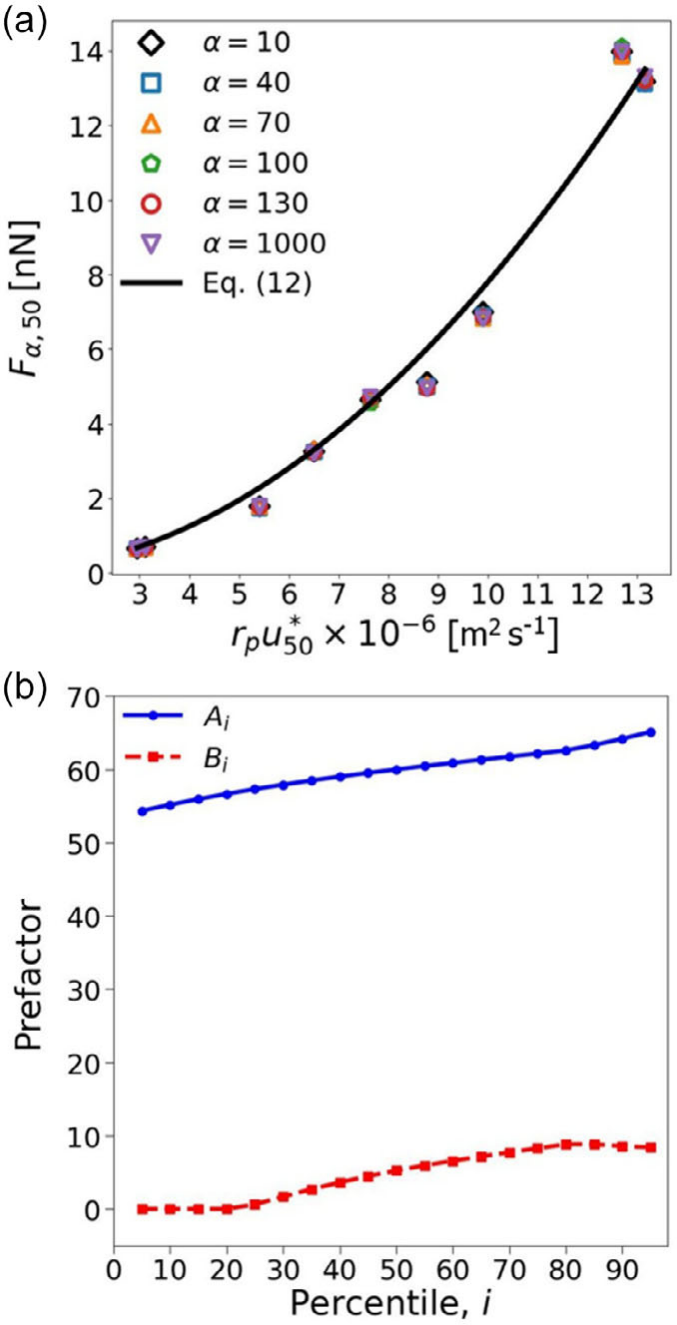
Drag‐lift decomposition at the median percentile and percentile‐resolved drag and lift coefficients. (a) Fit of the median normalized adhesion forces *F*_*α*,50_ using the drag‐lift informed scaling (Equation (12) ) for *α* = 10 (black diamonds), 40 (blue squares), 70 (orange triangles), 100 (green pentagons), 130 (red circles), and 1000 (purple inverted triangles). The data are well described by a dominant quadratic term (drag contribution), while the lift‐sensitive term contributes weakly and is less constrained. (b) Extracted coefficients *A*_*i*_ (drag‐related, blue) and *B*_*i*_ (lift‐related, red) across percentiles. The relatively stable values of *A*_*i*_ and the small, weakly constrained values of *B*_*i*_ indicate that rolling detachment is primarily drag‐controlled over the investigated percentile range.

The percentile‐resolved data were subsequently fitted using the drag‐lift‐informed expression given in Equation (12). Figure 3a shows that this formulation can provide a good description of the data, demonstrated by the case of the median percentile (*i* = 50). It yields a well‐constrained drag related coefficient *A*_50_ and a weakly constrained lift‐related coefficient *B*_50_ (Table 4) where *A*_50_ = 60 ± 1 and *B*_50_ = 5.2 ± 24.5. The fitted curve achieves a high coefficient of determination (*R* ^2^ = 0.98), indicating that the normalized adhesion forces are well captured by the aerodynamic torque scaling. Notably, the small magnitude and large uncertainty of *B*_50_ suggest that the lift contribution is weakly constrained and that the dominant dependence arises from the drag‐controlled term.

**TABLE 4:** Values for the coefficients *A*_i_ and *B*_i_ for various percentiles.

*i* (percentile)	*A*_i_	*B*_i_
10	55.16 ± 0.81	0.00 ± 24.58
25	57.30 ± 0.89	0.62 ± 24.81
50	59.98 ± 0.96	5.22 ± 24.54
75	62.14 ± 0.99	8.27 ± 23.15
90	64.16 ± 1.15	8.55 ± 25.65

Extending this analysis across percentiles (Figure 3b) reveals that *A*_*i*_ varies modestly (∼55–65), whereas *B*_*i*_ remains small and carries substantially larger uncertainty. This behavior of *B*_*i*_ suggests that the data have limited sensitivity to it. This parameter structure can be explained by the fact that the lift contribution to the rolling moment is weak. The result therefore provides quantitative support, within the present datasets and operating regime, for drag‐dominated rolling detachment.

For systems in which the particles are not fully immersed within the viscous sublayer, the present scaling should be regarded as outside its current range of validity. In such cases, the aerodynamic forcing and detachment pathway may include stronger lift‐off contributions, direct interaction with coherent turbulent structures, or nonlinear velocity profiles around the particle, and the RnR‐based inverse reconstruction would require corresponding modification.

The central objective of this analysis is to compare the fitted drag prefactors to the nominal RnR drag scaling (a numerical prefactor of 32 in the formulation of Equation (8) ). Thus, the following ratio is introduced: *λ*_*i*_ = 32*A*_*i*_^−1^, which quantifies the deviation between the strict model drag prefactor (32) and the effective resistance inferred from the data (*A*_*i*_). The resulting *λ*_*i*_ ∼ 0.49 − 0.59 indicates a systematic offset between the idealized near‐wall force model and the effective drag‐controlled resistance implied by the inverse reconstructions, plausibly reflecting combined effects of flow fluctuations, dataset to dataset protocol differences, and conditioning on percentiles.

### 3.4 Dimensionless Representation of Adhesion Resistance

Finally, to verify that the observed collapse of the data onto the curve in Figure 3a is not merely an artifact of fitting *F*_*α*,*i*_ versus *r*_*p*_ *u* ^*^, the full aerodynamic normalization is applied by introducing the dimensionless parameter

(13) $${\tilde{F}}_{\mathrm{adh},i}\;\equiv \frac{F_{adh,i}}{\alpha \rho r_{p}^{2}{u_{i}^{*}}^{2}}$$

which represents the moment‐equivalent adhesion resistance relative to the characteristic aerodynamic drag moment.

**FIGURE 4:**
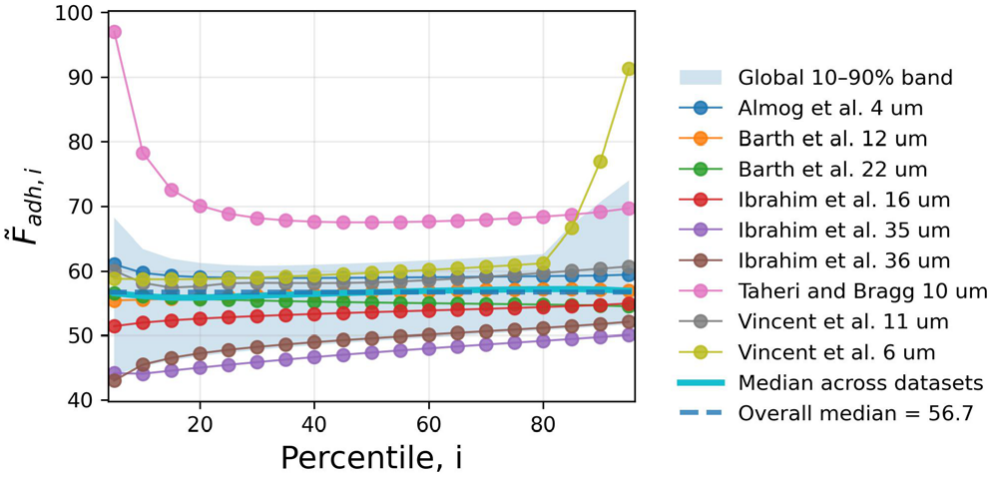
Dimensionless adhesion parameter ${\tilde{F}}_{\mathrm{adh},i}\;\equiv \;{\left(\alpha \rho r_{p}^{2}{u_{i}^{*}}^{2}\right)}^{‐1}$ across percentiles for all nine datasets. The shaded band represents the global 10%–90% interval of ${\tilde{F}}_{\mathrm{adh}}$. The collapse of the data into a narrow band demonstrates that aerodynamic normalization captures the dominant scaling of adhesion‐controlled detachment across diverse systems.

Figure 4 presents ${\tilde{F}}_{\mathrm{adh},i}$ across percentiles for all datasets. After normalization, the data collapse into a narrow band, shown by the shaded region in Figure 4, with only weak dependence on percentile. The majority of the values fall within a limited interval, indicating that the dominant scaling of adhesion‐controlled detachment is captured by the quadratic aerodynamic forcing embedded in Equation (13). The remaining spread primarily reflects experimental variability and weakly constrained percentile ranges. In particular, deviations at low percentiles for the Taheri and Bragg data and at high percentiles for Vincent et al. [42, 45], occur in regions where the original measurements provide limited information. Despite differences in particle size, materials, and experimental configurations, the collapse of ${\tilde{F}}_{\mathrm{adh},i}$ across datasets in a relatively narrow band (∼40–60) demonstrates that once aerodynamic forcing is properly accounted for, adhesion percentiles exhibit a nearly universal normalized resistance to rolling detachment.

Taken together, these results demonstrate that the apparent dependence of reconstructed adhesion forces on the geometric lever‐arm parameter *α* reflects a predictable compensatory scaling inherent to the rolling‐moment balance. Once normalized by *α*, adhesion percentiles exhibit a near quadratic aerodynamic scaling governed predominantly by drag. The weak contribution of lift further supports a drag dominated detachment mechanism. Importantly, the identifiable dimensionless adhesion parameters across nine independent datasets indicates that the IbO framework yields physically consistent adhesion statistics that are robust to variations in particle size, material combination, and prescribed values of *α*. This establishes a practical modeling constraint: predictive resuspension behavior can be anchored to drag‐based aerodynamic scaling, with *α* acting as a geometric scaling parameter rather than additional degree of freedom.

## 4 Conclusion

This study introduces a data‐driven inverse approach that extracts effective adhesion force statistics directly from particle resuspension measurements by coupling the IbO algorithm with the RnR model into which the beta distribution was integrated. By reconstructing adhesion distributions from measured resuspension kinetics, the introduced approach reduces reliance on prescribed adhesion variability while preserving consistency with aerodynamic forcing and rolling‐moment balance.

A central finding is that inferred adhesion percentiles correlate systematically with the combined aerodynamic parameter $r_{p}u^{*}$, yielding exponent values within the range of those to which the drag and lift forces are raised in the original RnR formulations. When normalized by the lever‐arm ratio $\alpha =r_{p}/a$, the reconstructed adhesion forces obtained under different prescribed *α* values exhibit a pronounced minimization of scatter. This reduction arises from the linear role of *α* in the rolling‐moment balance. Therefore, it serves as a self‐consistency check of the inverse reconstruction. The residual variations following normalization provide physical insights on percentile‐dependent detachment kinetics and dataset‐specific dynamics.

Comparison between the fitted drag‐related coefficient *A*_*i*_ and the nominal drag prefactor in the classical RnR model [30] reveals systematic deviations. Defining $\lambda =32A_{i}^{-1}$ quantifies this departure from idealized deterministic rolling. The consistently subunity values of *λ* indicate enhanced effective resistance relative to nominal drag‐only scaling, reflecting the combined influence of geometric variability, flow intermittency, and stochastic detachment processes. Importantly, *λ* should be interpreted not as a correction to the drag law itself, but as a compact indicator of the difference between idealized torque balance and experimentally inferred rolling resistance.

Beyond the dimensional scaling, the introduction of an aerodynamic normalized adhesion parameter ${\tilde{F}}_{\mathrm{adh},i}$ reveals that adhesion‐controlled detachment operates within a relatively narrow dimensionless range across multiple datasets, particle sizes, and percentiles. The near stability of ${\tilde{F}}_{\mathrm{adh},i}$ demonstrates that once aerodynamic forcing and rolling geometry are properly accounted for, adhesion percentiles collapse onto a consistent regime. This dimensionless metric therefore provides a robust basis for benchmarking resuspension behavior across heterogeneous experimental conditions.

While *α* is treated here as a prescribed geometric parameter, the results underscore its pivotal role in mediating the inferred adhesion statistics. The inverse reconstruction does not eliminate geometric uncertainty in the rolling lever‐arm *a*; rather, it isolates the percentile‐resolved moment‐equivalent resistance *F*_*α*,*i*_, the mechanically relevant quantity governing rolling detachment.

A limitation of the present approach is that, although several values of *α* were examined, where *α* was prescribed for each individual reconstruction rather than independently inferred. In real particle‐surface systems, this parameter is expected to exhibit a distribution associated with particle geometry, surface roughness, asperity configuration, and local contact mechanics. In addition, the reconstructed adhesion forces should be interpreted as effective moment‐equivalent adhesion resistances associated with rolling detachment, rather than as direct pull‐off adhesion forces. The present analysis is also restricted to datasets satisfying the underlying assumptions of the RnR formulation, particularly particles fully immersed within the viscous sublayer and sparse deposits for which particle–particle interactions are limited. Finally, the analyzed datasets mainly involve nearly spherical particles, whereas environmental and industrial particles are often irregular, rough, or present in multilayer deposits. Extending the framework to incorporate distributions of *α*, particle‐shape effects, surface roughness, and collective or collision‐assisted resuspension therefore represents an important direction for future work. Such extensions may allow simultaneous inference of adhesion and contact‐geometry statistics directly from resuspension data.

Overall, this work demonstrates that resuspension measurements, when interpreted through an inverse algorithm, can effectively provide adhesion force statistics and their aerodynamic scaling. By clarifying the interplay between drag‐controlled forcing, rolling mechanics, and stochastic escape, the presented approach establishes a physically grounded and extensible pathway toward predictive resuspension modeling without direct adhesion force measurements.

## Author Contributions

**Roy Almog:** conceptualization, writing – original draft, visualization, software, methodology, investigation, formal analysis. **Ziv Klausner:** writing – review & editing, writing – original draft, visualization, validation, supervision, funding acquisition. **Ronen Berkovich:** conceptualization, writing – review & editing, writing – original draft, visualization, supervision, funding acquisition.

## Funding

This work was supported by the Pazy Foundation, grant ID 133‐2020.

## Conflicts of Interest

The authors declare no conflicts of interest.

## Supporting information

Supplementary Material

## Data Availability

All data related to this project are included in the main text.

## References

[1] D. Đorđević , Z. Vukmirović , I. Tošić , and M. Unkašević , “Contribution of Dust Transport and Resuspension to Particulate Matter Levels in the Mediterranean Atmosphere,” Atmospheric Environment 38 (2004): 3637.

[2] H.‐C. Kung , W.‐C. Lin , B.‐W. Huang , J. K. Mutuku , and G.‐P. Chang‐Chien , “Techniques for Suppressing Mineral Dust Aerosol from Raw Material Stockpiles and Open Pit Mines: A Review,” Aerosol and Air Quality Research 24 (2024): 230166.

[3] L. Gradoń , “Resuspension of Particles from Surfaces: Technological, Environmental and Pharmaceutical Aspects,” Advanced Powder Technology 20 (2009): 17.

[4] R. Przekop , K. Grzybowski , and L. Gradoń , “Energy‐Balanced Oscillatory Model for Description of Particles Deposition and Re‐Entrainment on Fiber Collector,” Aerosol Science and Technology 38 (2004): 330.

[5] Y. Qian , K. Willeke , V. Ulevicius , and S. A. Grinshpun , “Particle Reentrainment from Fibrous Filters,” Aerosol Science and Technology 27 (1997): 394.

[6] W. De Vos , W. Nicol , and E. Du Toit , “Entrainment Behaviour of High‐Density Geldart a Powders with Different Shapes,” Powder Technology 190 (2009): 297.

[7] S. R. Haines , R. I. Adams , B. E. Boor , et al., “Ten Questions concerning the Implications of Carpet on Indoor Chemistry and Microbiology,” Building and Environment 170 (2020): 106589. 10.1016/j.buildenv.2019.106589 PMC7017391 32055099

[8] P. Salimifard , D. Rim , C. Gomes , P. Kremer , and J. D. Freihaut , “Resuspension of Biological Particles from Indoor Surfaces: Effects of Humidity and Air Swirl,” Science of the Total Environment 583 (2017): 241. 28117152 10.1016/j.scitotenv.2017.01.058

[9] M. Corn and F. Stein , “Re‐Entrainment of Particles from a Plane Surface,” American Industrial Hygiene Association Journal 26 (1965): 325. 5854669 10.1080/00028896509342739

[10] C. Henry and J.‐P. Minier , “Progress in particle resuspension from rough surfaces by turbulent flows,” Progress in Energy and Combustion Science 45 (2014): 1.

[11] G. Ziskind , M. Fichman , and C. Gutfinger , “Resuspension of Particulates from Surfaces to Turbulent Flows—Review and Analysis,” Journal of Aerosol Science 26 (1995): 613.

[12] G. Ziskind , “Particle resuspension from surfaces: revisited and re‐evaluated,” Reviews in Chemical Engineering 22 (2006): 1.

[13] C. Henry and J.‐P. Minier , “A Stochastic Approach for the Simulation of Particle Resuspension from rough Substrates: Model and Numerical Implementation,” Journal of Aerosol Science 77 (2014): 168.

[14] D. Ben Shlomo , R. Almog , Z. Klausner , E. Fattal , and R. Berkovich , “Introducing Surface Roughness in Adhesion for Stochastic and Rock‘n’Roll Models to Describe Particle Resuspension in Turbulent Flows,” Surfaces and Interfaces 48 (2024): 104321.

[15] C. Henry , J.‐P. Minier , and S. Brambilla , “Particle Resuspension: Challenges and Perspectives for Future Models,” Physics Reports 1007 (2023): 1.

[16] A. H. Ibrahim and P. F. Dunn , “Effects of Temporal Flow Acceleration on the Detachment of Microparticles from Surfaces,” Journal of Aerosol Science 37 (2006): 1258.

[17] C. Habchi , K. Ghali , and N. Ghaddar , “Coupling CFD and Analytical Modeling for Investigation of Monolayer Particle Resuspension by Transient Flows,” Building and Environment 105 (2016): 1.

[18] J. G. Benito , K. A. V. Aracena , R. O. Uñac , A. M. Vidales , and I. Ippolito , “Monte Carlo Modelling of Particle Resuspension on a Flat Surface,” Journal of Aerosol Science 79 (2015): 126.

[19] D. A. Braaten , U. K. T. Paw , and R. H. Shaw , “Particle Resuspension in a Turbulent Boundary Layer‐Observed and Modeled,” Journal of Aerosol Science 21 (1990): 613.

[20] A. H. Ibrahim , P. F. Dunn , and R. M. Brach , “Microparticle Detachment from Surfaces Exposed to Turbulent Air Flow: Controlled Experiments and Modeling,” Journal of Aerosol Science 34 (2003): 765.

[21] Y. Kim , A. Gidwani , B. E. Wyslouzil , and C. W. Sohn , “Source Term Models for Fine Particle Resuspension from Indoor Surfaces,” Building and Environment 45 (2010): 1854.

[22] B. Nasr , G. Ahmadi , A. R. Ferro , and S. Dhaniyala , “Overview of Mechanistic Particle Resuspension Models: Comparison with Compilation of Experimental Data,” Journal of Adhesion Science and Technology 33 (2019): 2631.

[23] G. Ziskind , M. Fichman , and C. Gutfinger , “Adhesion Moment Model for Estimating Particle Detachment from a Surface,” Journal of Aerosol Science 28 (1997): 623.

[24] Q. Sun , S. Yu , and W. Peng , “Experimental and Mechanistic Study of Dispersed Micrometer‐Sized Particle Resuspension in a Square Straight Duct with rough Walls,” Particuology 83 (2023): 101.

[25] Q. Sun , S. He , B. Liu , L. Shi , and W. Peng , “Study of Particle Dynamic Resuspension in a Recirculation Flow Using Large‐Eddy Simulations,” Powder Technology 428 (2023): 118832.

[26] L. Biasi , A. de los Reyes , M. W. Reeks , and G. F. de Santi , “Use of a Simple Model for the Interpretation of Experimental Data on Particle Resuspension in Turbulent Flows,” Journal of Aerosol Science 32 (2001): 1175.

[27] J. G. Benito , R. O. Uñac , A. M. Vidales , and I. Ippolito , “Kinetic Programmed Resuspension – KPR Technique,” Journal of Aerosol Science 122 (2018): 21.

[28] K. L. Johnson , K. Kendall , and R. Roberts , Proceedings of the royal society of London. A. mathematical and physical sciences (1971), 301.

[29] B. V. Derjaguin , V. M. Muller , and Y. P. Toporov , “Effect of Contact Deformations on the Adhesion of Particles,” Journal of Colloid and Interface Science 53 (1975): 314.

[30] M. W. Reeks and D. Hall , “Kinetic Models for Particle Resuspension in Turbulent Flows: Theory and Measurement,” Journal of Aerosol Science 32 (2001): 1.

[31] M. N. Rush , S. Brambilla , S. Speckart , G. A. Montaño , and M. J. Brown , “Glass‐Particle Adhesion‐Force‐Distribution on Clean (laboratory) and Contaminated (outdoor) Surfaces,” Journal of Aerosol Science 123 (2018): 231.

[32] H. Zhou , M. Götzinger , and W. Peukert , “The Influence of Particle Charge and Roughness on Particle–substrate Adhesion,” Powder Technology 135‐136 (2003): 82.

[33] O. Laitinen , K. Bauer , J. Niinimäki , and U. A. Peuker , “Validity of the Rumpf and the Rabinovich Adhesion Force Models for Alumina Substrates with Nanoscale Roughness,” Powder Technology 246 (2013): 545.

[34] S. Brambilla , S. Speckart , M. N. Rush , G. A. Montano , and M. J. Brown , “Glass Particle Resuspension from a Contaminated (dirty) Glass Surface,” Journal of Aerosol Science 123 (2018): 122.

[35] R. Almog , V. Babin , R. Berkovich , and Z. Klausner , “The Beta Distribution as a Generalized Statistical Model for Adhesion Force,” Journal of Colloid and Interface Science 705 (2026): 139451. 41289868 10.1016/j.jcis.2025.139451

[36] P. Fillingham , K. Kottapalli , X. Zhan , and I. V. Novosselov , “Characterization of Adhesion Force in Aerodynamic Particle Resuspension,” Journal of Aerosol Science 128 (2019): 89.

[37] J. Feng , C. Wang , Y. Zhang , et al., “Particle Resuspension from a Flow‐Induced Fluttering Flexible Substrate,” Powder Technology 415 (2023): 118163.

[38] H. Z. Ting , Y. Yang , Z. F. Tian , T. Carageorgos , and P. Bedrikovetsky , “Detachment of Inclined Spheroidal Particles from Flat Substrates,” Powder Technology 427 (2023): 118754.

[39] D. Yu , J.‐L. Lin , and J.‐H. Xie , “Investigation on the Influence of Vortex Generator on Particle Resuspension,” Particuology 86 (2024): 126.

[40] S. Brambilla and M. J. Brown , “Impact of the Adhesion‐Force Lever‐Arm “a” on the Rock ‘n’ Roll Resuspension Model and How to Compute It from Contact Mechanics,” Journal of Aerosol Science 143 (2020): 105525.

[41] F. Zhang , M. Reeks , and M. Kissane , “Particle Resuspension in Turbulent Boundary Layers and the Influence of Non‐Gaussian Removal Forces,” Journal of Aerosol Science 58 (2013): 103.

[42] J. C. Vincent , J. Hill , M. D. Walker , S. A. Smith , S. E. Smith , and N. E. Cant , “Towards a Predictive Capability for the Resuspension of Particles through Extension and Experimental Validation of the Biasi Implementation of the “Rock‘n’Roll” Model,” Journal of Aerosol Science 137 (2019): 105435.

[43] A. Rondeau , S. Peillon , A. M. Vidales , et al., “Evidence of Inter‐Particles Collision Effect in Airflow Resuspension of Poly‐Dispersed Non‐Spherical Tungsten Particles in Monolayer Deposits,” Journal of Aerosol Science 154 (2021): 105735.

[44] R. Almog , D. Ben Shlomo , S. Sevilia , et al., “Optimized Reconstruction of Adhesion Force Distribution from Resuspension Measurements Using the Rock‘n’Roll Model,” Journal of Aerosol Science 185 (2025): 106539.

[45] M. Taheri and G. M. Bragg , “A Study of Particle Resuspension in a Turbulent Flow Using a Preston Tube,” Aerosol Science and Technology 16 (1992): 15.

[46] T. Barth , J. Preuß , G. Müller , and U. Hampel , “Single Particle Resuspension Experiments in Turbulent Channel Flows,” Journal of Aerosol Science 71 (2014): 40.

[47] N. L. Johnson and S. Kotz , Continuous Univariate Distributions (John Wiley & Sons, 1970).

[48] S. Biggs , R. G. Cain , R. R. Dagastine , and N. W. Page , “Direct Measurements of the Adhesion between a Glass Particle and a Glass Surface in a Humid Atmosphere,” Journal of Adhesion Science and Technology 16 (2002): 869.

