# Supplementary material for: Moment‐Equivalent Adhesion Statistics Inferred From Particle Resuspension Kinetics
Source: Small Sci. 2026 Aug 11;6(8):e70361. doi: 10.1002/smsc.70361 (PMC13489309; doi:10.1002/smsc.70361)
Supplement: Supplementary file 1 [file SMSC-6-e70361-s001.pdf]

Supporting Information

Moment-Equivalent Adhesion Statistics Inferred from Particle Resuspension Kinetics

Roy Almog, Ziv Klausner, and Ronen Berkovich**

R. Almog, R. Berkovich

Department of Chemical Engineering, Ben-Gurion University of the Negev, Beer-Sheva 8410501, Israel.

Z. Klausner

Department of Applied Mathematics, Israel Institute for Biological Research, Ness-Ziona 7410001, Israel.

* E-mail: zivk@iibr.gov.il

R. Berkovich

Ilze Katz Institute for Nanoscience and Technology, Ben-Gurion University of the Negev, Beer-Sheva 8410501, Israel.

* E-mail: berkovir@bgu.ac.il

1. Sensitivity and practical identifiability of the beta-based reconstruction

The inverse-by-optimization (IbO) algorithm used in this study reconstructs an adhesion-force distribution by identifying the beta-distribution parameters ($p,q,\ F_{min},\ F_{max}$) that best reproduce the measured resuspension curve through the RnR model. Because inverse problems can be non-unique, it is important to examine whether different beta-parameter sets can produce similar adhesion-force distributions and similar resuspension predictions.

This analysis distinguishes between two levels of identifiability. The first is the identifiability of the individual beta parameters. The second is the practical identifiability of the reconstructed adhesion-force CDF and of the adhesion-force percentiles extracted from it. The scaling analysis in the main manuscript uses only the latter quantities

As a first demonstration, Table S1 lists three different beta-parameter sets. Although the numerical values of $p,q,\ F_{min},\ F_{max}$ differ, the corresponding adhesion-force CDFs nearly overlap, as shown in Fig. S1a. Consequently, the adhesion-force percentiles extracted from these CDFs are nearly identical. When these distributions are implemented in the RnR model using the same remaining parameters,r_p = 4 μm, r_p/a = 50, and $\sqrt{f^2} = 0.2\langle F\rangle$, and τ = 1 s, the resulting resuspension curves are also nearly identical, as shown in Fig. S1b.

Table S1. Three sets of beta-distribution parameters that yield almost identical CDFs.

Case	p	q	F_{min} [nN]	F_{max} [nN]
1	3.7	7.6	60	2000
2	4.5	10.2	6	2250
3	3.0	5.6	120	1800

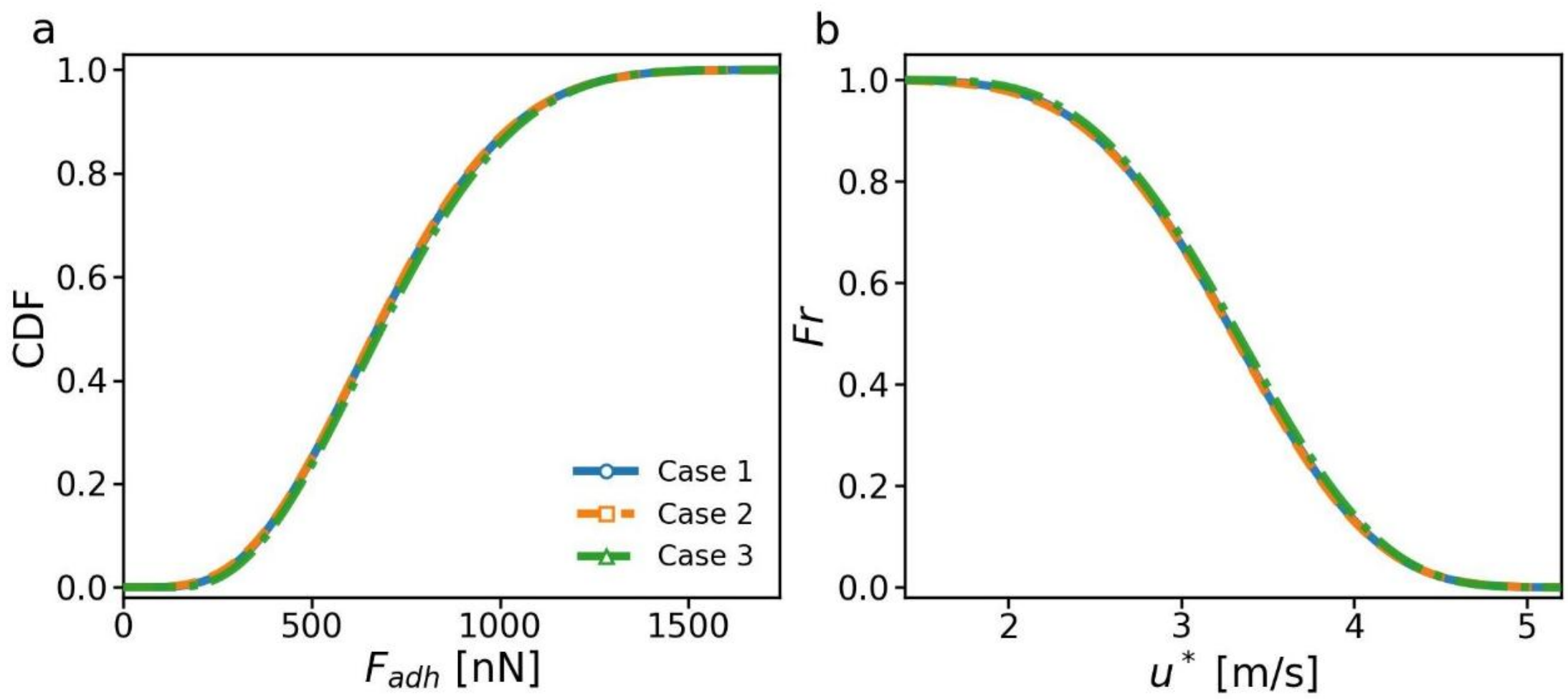

Figure S1. Demonstration of parameter non-uniqueness. Different beta-parameter sets listed in Table S1 produce nearly identical adhesion-force CDFs (a). When these distributions are implemented in the RnR model using the same remaining model parameters, they also produce nearly identical predicted remaining-fraction curves, $F_r(u^*)$ (b).

The inverse reconstruction problem was then considered, in which adhesion-force distributions are inferred from resuspension data. The analysis was performed using the Barth et al. dataset for 12.5 μm-radius GBM particles, with r_p/a = 100. The reference case corresponds to the beta distribution selected in the main analysis, which provided the lowest RMSE value of 0.0129. Alternative beta-parameter sets with similarly low RMSE values were then identified using the same IbO procedure.

Table S2 reports the resulting beta parameters, RMSE values, and relative differences between selected adhesion-force percentiles and the reference case. The relative percentile difference is defined as $D_{F,i} = 100\left(F_{i,case} - F_{i,ref}\right)/F_{i,ref}$, where F_i is the i-th adhesion-force percentile.

Table S2. IbO-derived beta-parameter sets for Barth et al. 12.5 μm GBM particles, with RMSE values and percentile differences from the reference case.

**Set**	p	q	F_{min} **[nN]**	F_{max} **[nN]**	**RMSE**	$D_{F,5}$ **[%]**	$D_{F,25}$ **[%]**	$D_{F,50}$ **[%]**	$D_{F,75}$ **[%]**	$D_{F,95}$ **[%]**
**Ref.**	2.2	2.8	42	94	0.0129	0	0	0	0	0
**2**	2.5	3.2	42	94	0.0131	1.5	0.8	0.0	-0.8	-1.3
**3**	2.1	2.8	42	94	0.0134	-1.0	-1.2	-1.1	-0.8	-0.4
**4**	1.9	2.8	45	94	0.0135	2.5	0.3	-0.5	-0.7	-0.5
**5**	2.5	2.4	39	89	0.0138	-1.8	0.0	0.2	-0.4	-2.0

The corresponding RnR-predicted resuspension curves are shown in Fig. S2a, together with the measured Barth et al. data. All reconstructed curves closely reproduce the measured sigmoidal trend, with only minor deviations in the transition region. The corresponding adhesion-force CDFs, shown in Fig. S2b, also strongly overlap. The maximum percentile deviation among the examined cases is 2.5%, and most deviations are close to or below 1%.

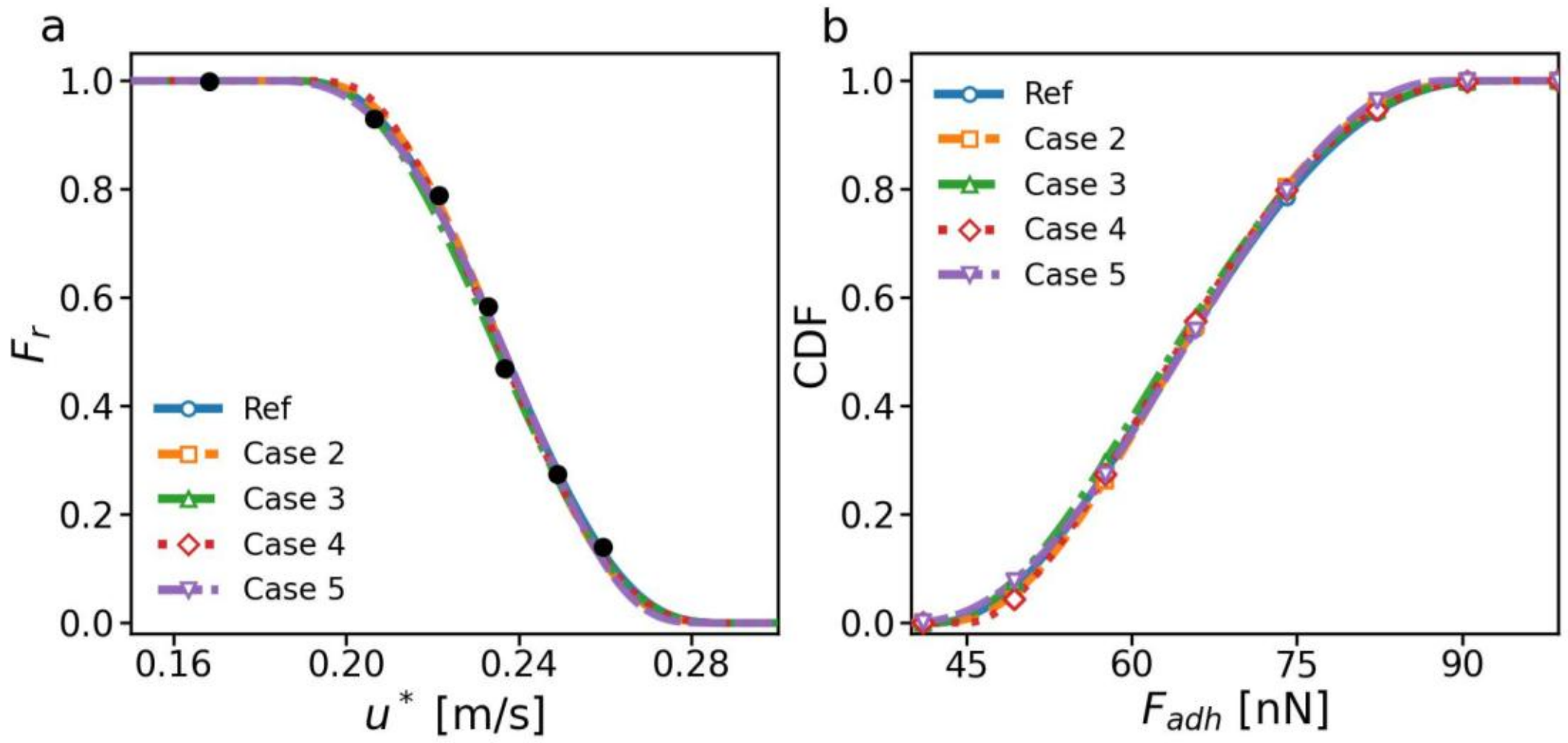

Figure S2. Inverse-reconstructed results for the Barth et al. 12.5 µm-radius GBM particles. (a) Measured resuspension data and modeled RnR curves. Black circles represent the experimental data, and colored curves represent the modeled curves obtained from the beta-parameter sets listed in Table S2. (b) Corresponding reconstructed adhesion-force CDFs.

These results indicate that the individual beta parameters are not uniquely identifiable. Changes in F_{min} and F_{max} can be compensated by changes in p and q, producing similar CDFs and similar RnR predictions. Therefore, the beta parameters should not be interpreted in isolation. The relevant reconstructed quantity is the complete adhesion-force CDF, and the percentile-resolved adhesion forces extracted from it. The scaling analysis in the main manuscript is consequently based on these percentiles rather than on the individual beta parameters.

S2. Comparison between beta and lognormal adhesion-force reconstructions

To directly compare the bounded beta formulation with the conventional lognormal representation, the IbO procedure was repeated using both adhesion-force distributions within the same RnR-based framework. For each experimental dataset, all RnR model inputs and dataset-specific parameters were kept identical between the two reconstructions. Only the functional form of the adhesion-force distribution and its fitted parameters were changed.

For the beta reconstruction, the adhesion-force distribution was described by the shape parameters $p$and q, together with the finite bounds $F_{\min}$ and $F_{\max}$. For the lognormal reconstruction, the adhesion-force distribution was described using the conventional spread and reduction parameters. For each distribution type, the optimal parameter set was defined as the one that minimized the root mean squared error, RMSE, between the measured remaining fraction and the model-predicted remaining fraction over the experimentally measured range of friction velocities.

The RMSE values reported in Table S3 quantify only the agreement between the measured and fitted resuspension curves. The adhesion-force CDFs shown in Figures S3–S11 are the distributions corresponding to the parameter sets that minimized the resuspension-curve error. Therefore, the CDFs should be interpreted as qualitative comparisons between the inferred adhesion-force distributions, rather than as independent quantitative error measurements. This distinction is important because independent experimental adhesion-force CDFs are not available for these literature datasets.

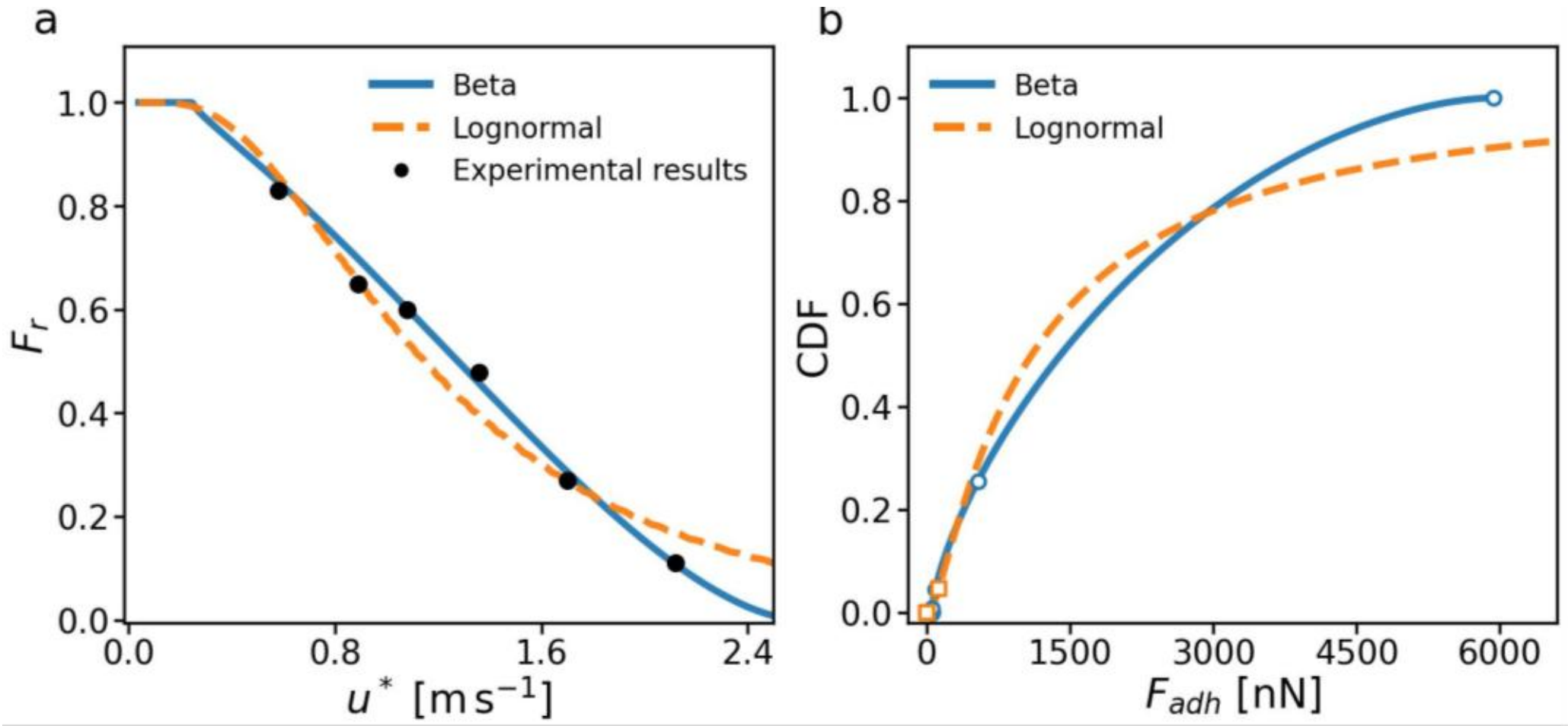

Figure S3. Comparison between beta and lognormal reconstructions for Taheri and Bragg,[1] r_p = 10 μm. (a) Measured and fitted remaining-fraction curves as a function of friction velocity. (b) Adhesion-force CDFs corresponding to the optimal beta and lognormal parameter sets.

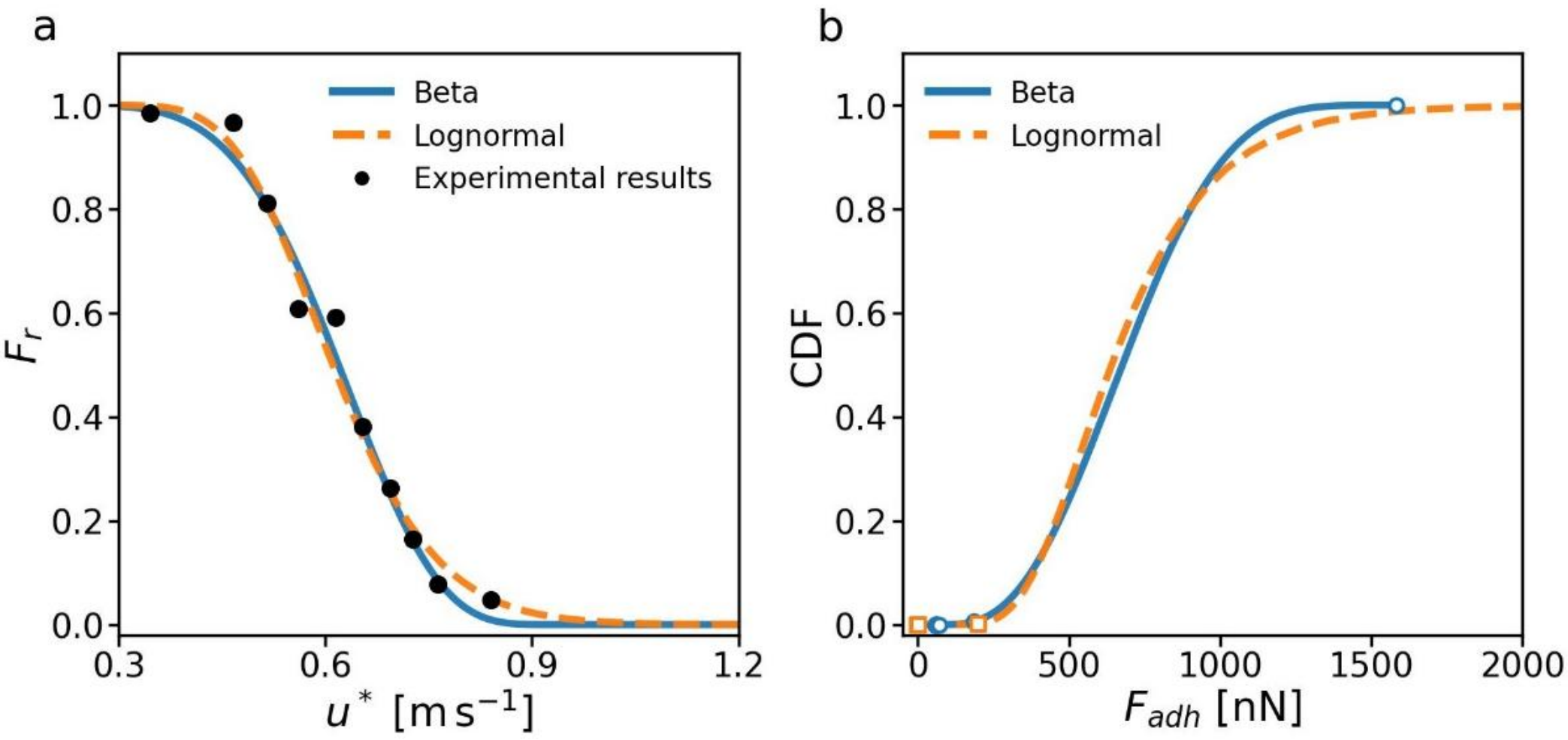

Figure S4. Comparison between beta and lognormal reconstructions for Ibrahim et al.,[2] r_p = 16 μm. (a) Measured and fitted remaining-fraction curves as a function of friction velocity. (b) Adhesion-force CDFs corresponding to the optimal beta and lognormal parameter sets.

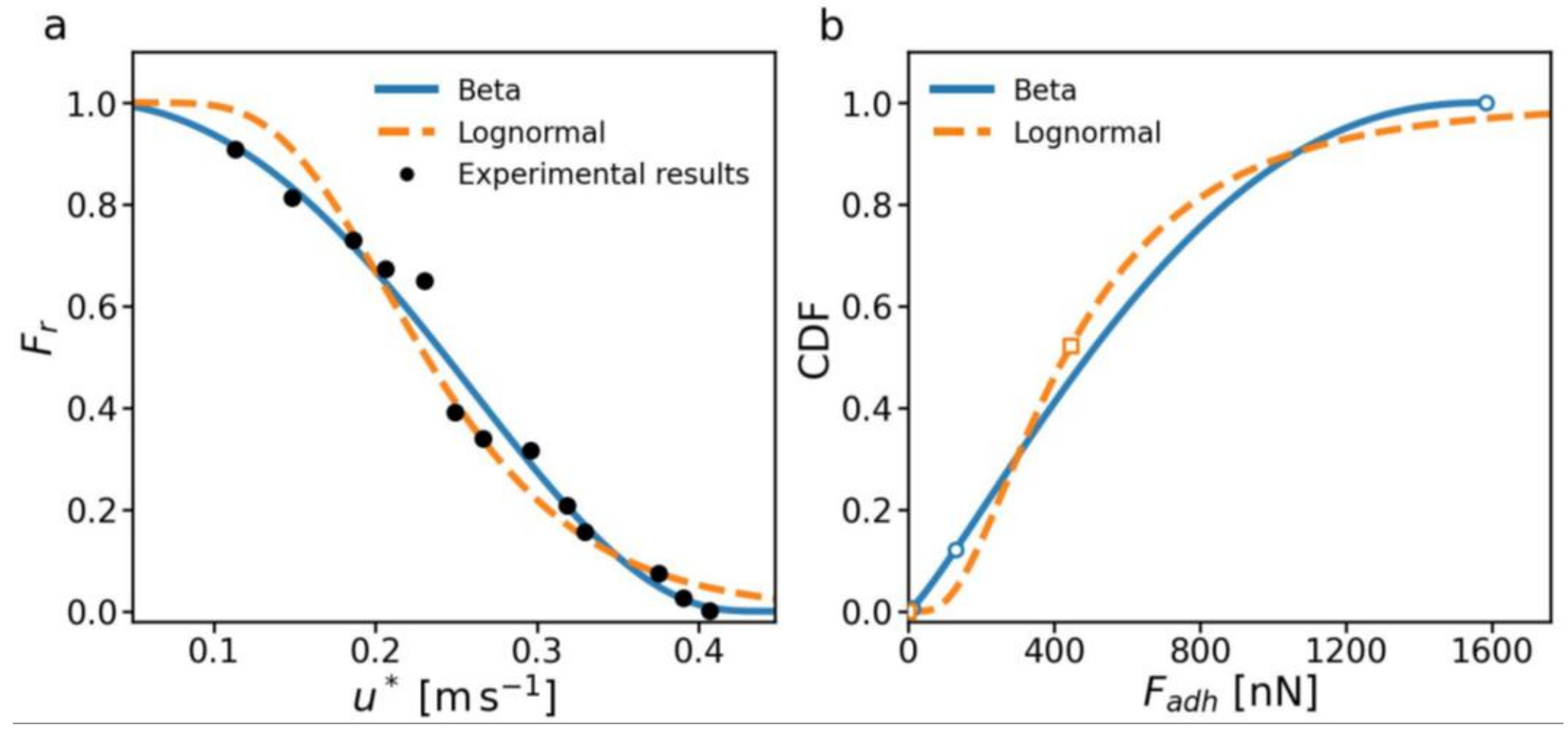

Figure S5. Comparison between beta and lognormal reconstructions for Ibrahim et al.,[2] r_p = 36 μm. (a) Measured and fitted remaining-fraction curves as a function of friction velocity. (b) Adhesion-force CDFs corresponding to the optimal beta and lognormal parameter sets.

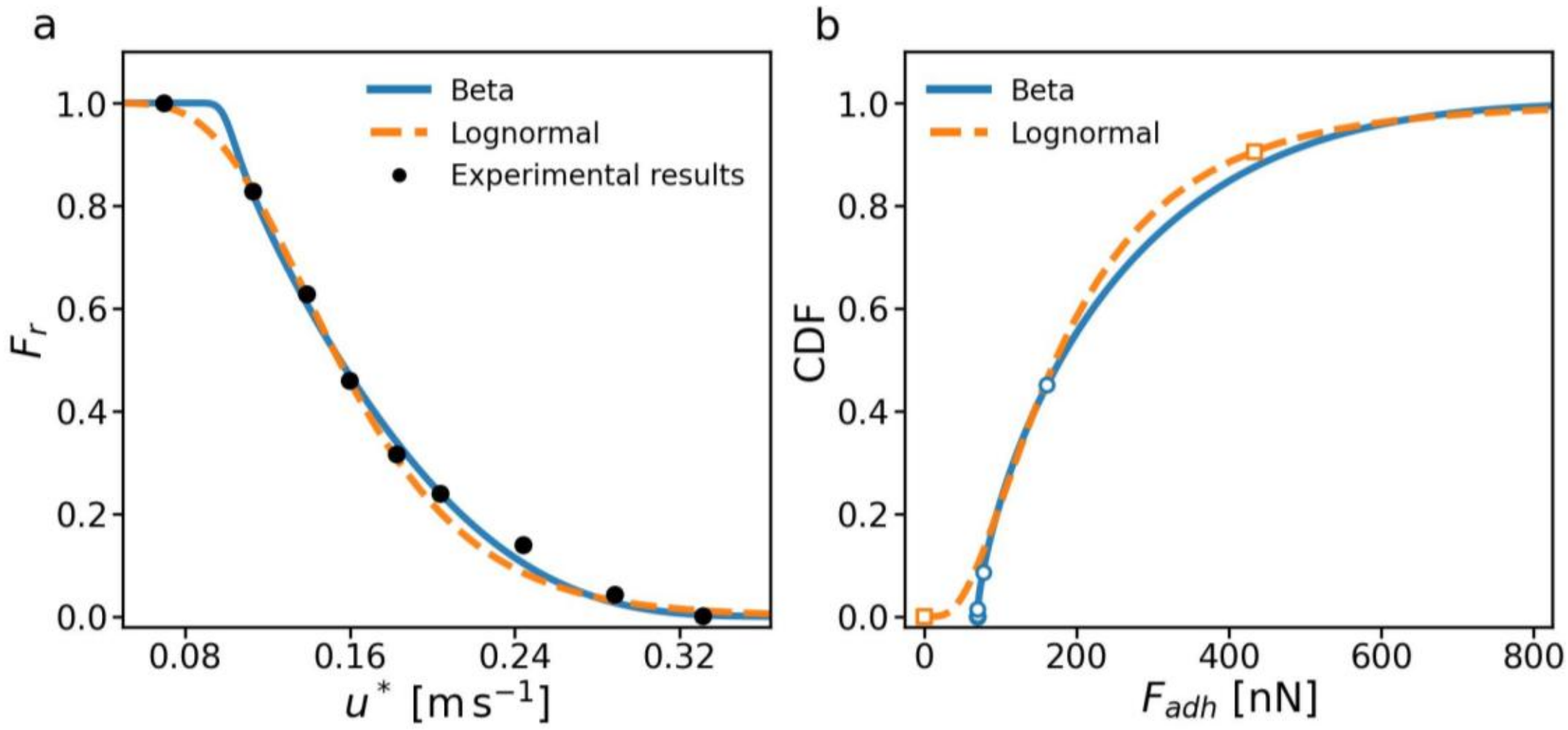

Figure S6. Comparison between beta and lognormal reconstructions for Ibrahim et al.,[2] r_p = 35 μm. (a) Measured and fitted remaining-fraction curves as a function of friction velocity. (b) Adhesion-force CDFs corresponding to the optimal beta and lognormal parameter sets.

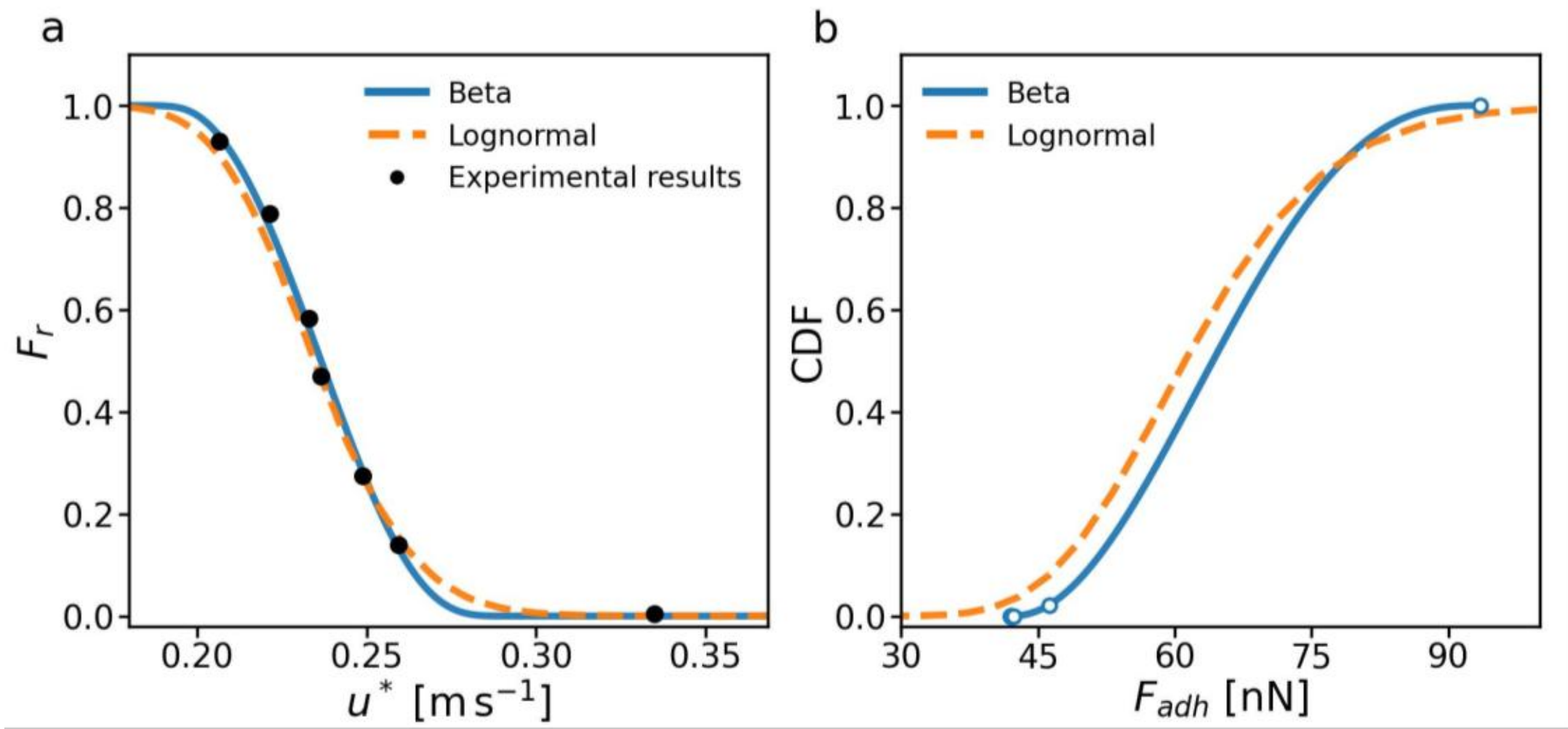

Figure S7. Comparison between beta and lognormal reconstructions for Barth et al.,[3] r_p = 12.5 µm. (a) Measured and fitted remaining-fraction curves as a function of friction velocity. (b) Adhesion-force CDFs corresponding to the optimal beta and lognormal parameter sets.

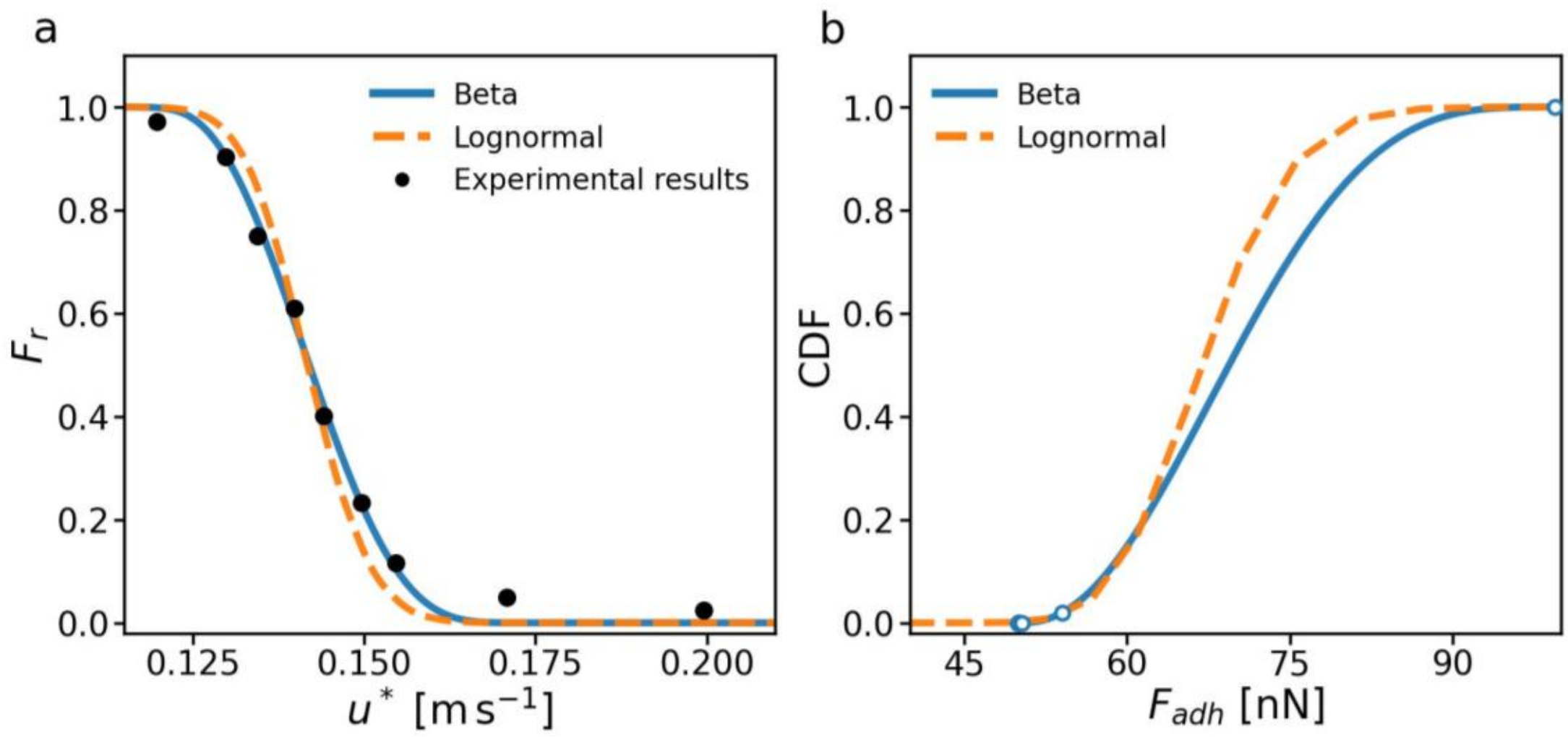

Figure S8. Comparison between beta and lognormal reconstructions for Barth et al.,[3] r_p = 22 µm. (a) Measured and fitted remaining-fraction curves as a function of friction velocity. (b) Adhesion-force CDFs corresponding to the optimal beta and lognormal parameter sets.

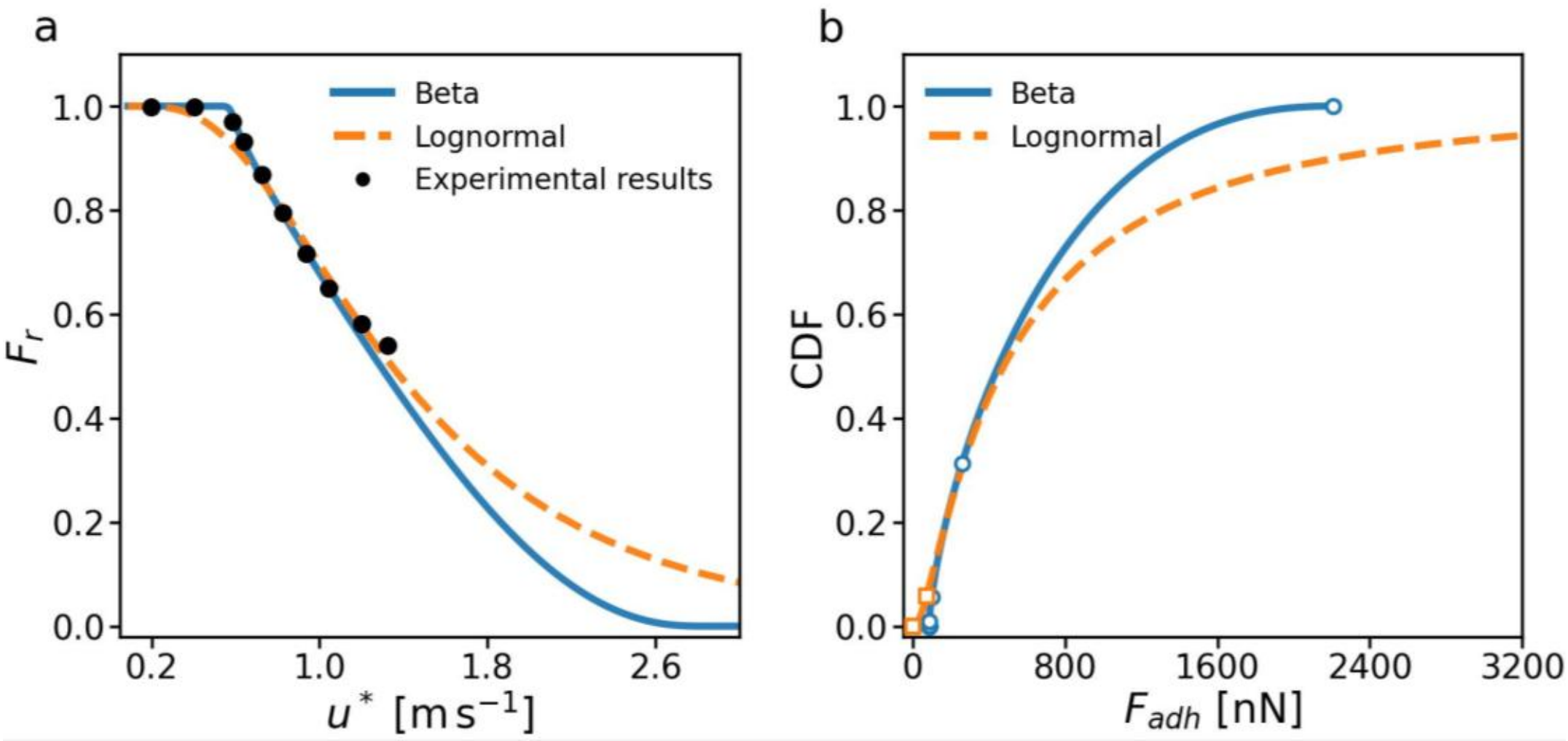

Figure S9. Comparison between beta and lognormal reconstructions for Vincent et al.,[4] r_p = 6 µm. (a) Measured and fitted remaining-fraction curves as a function of friction velocity. (b) Adhesion-force CDFs corresponding to the optimal beta and lognormal parameter sets.

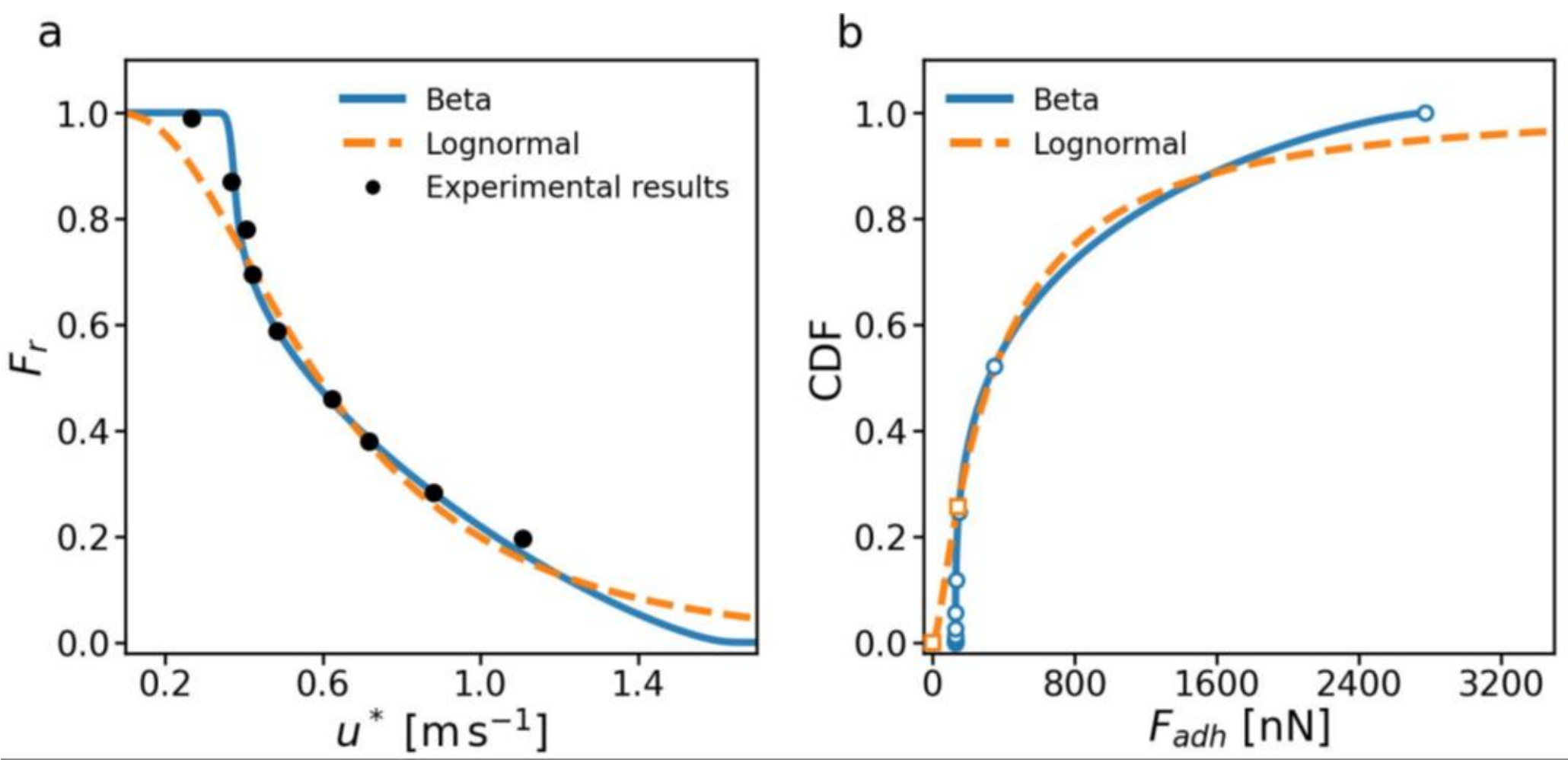

Figure S10. Comparison between beta and lognormal reconstructions for Vincent et al.,[4] r_p = 11.5 µm. (a) Measured and fitted remaining-fraction curves as a function of friction velocity. (b) Adhesion-force CDFs corresponding to the optimal beta and lognormal parameter sets.

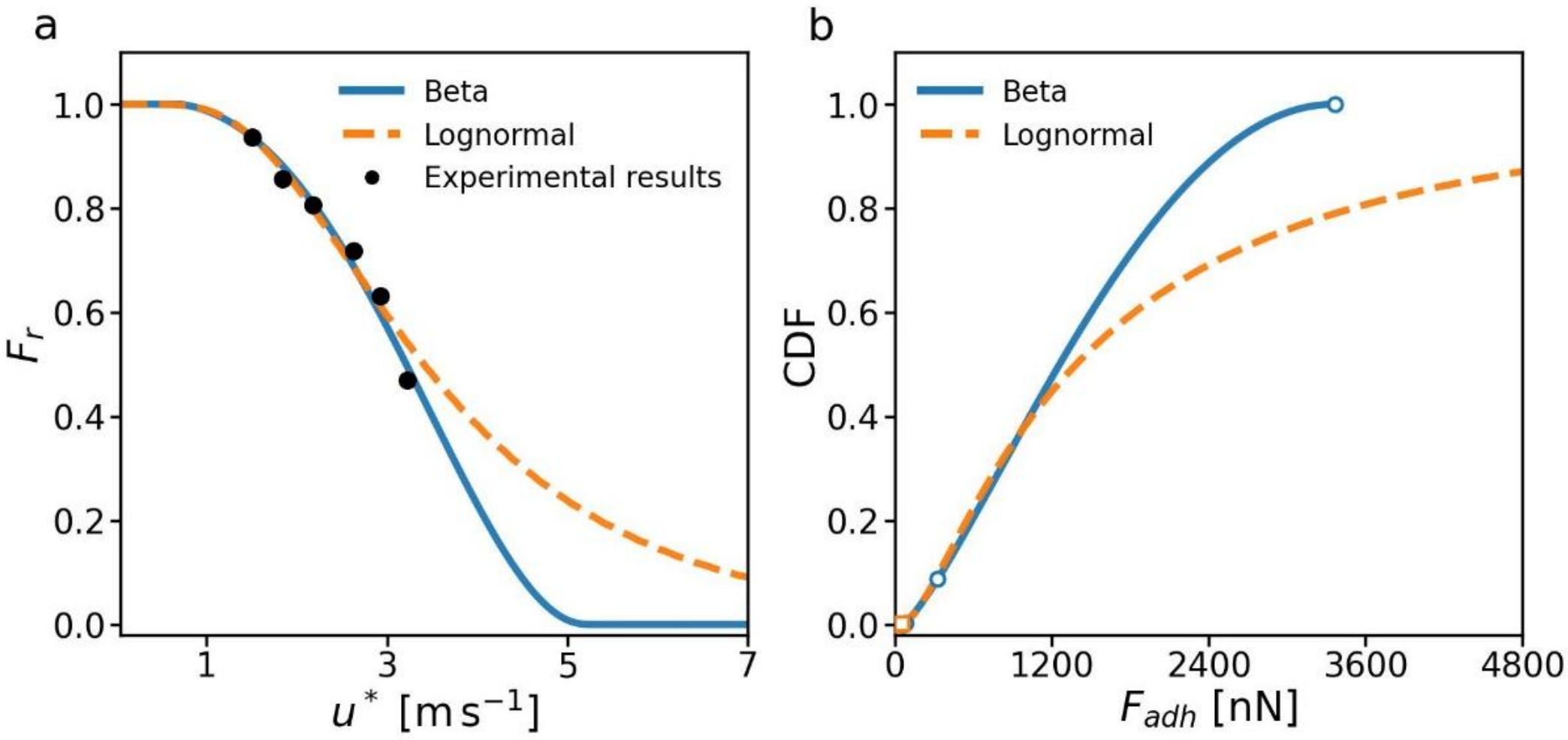

Figure S11. Comparison between beta and lognormal reconstructions for Almog et al.,[5] r_p = 4 µm. (a) Measured and fitted remaining-fraction curves as a function of friction velocity. (b) Adhesion-force CDFs corresponding to the optimal beta and lognormal parameter sets.

Table S3 summarizes the fitted beta and lognormal parameters and their corresponding RMSE values. For the nine datasets examined, the beta reconstruction produced lower RMSE values than the lognormal reconstruction in all cases. The mean RMSE decreased from approximately 0.0291 for the lognormal fits to approximately 0.0173 for the beta fits. The standard deviation of the RMSE for the beta fits was halved (0.057) compared to the standard deviation of the lognormal fits (0.0118). The RMSE improvement was substantial in several datasets, including Taheri and Bragg, Ibrahim et al. for r_p = 36 µm, Barth et al. for r_p = 12.5 µm, and Vincent et al. for r_p = 11.5 µm. In these cases, visible differences are observed both in the fitted resuspension curves and in the corresponding adhesion-force CDFs.

In other datasets, the beta and lognormal reconstructions were more similar. For example, for Ibrahim et al. with r_p = 16 µm and r_p = 35 µm, the two fitted resuspension curves are close over most of the measured velocity range, and the corresponding RMSE values differ only slightly. These cases indicate that the lognormal distribution can still provide an adequate representation when the inferred adhesion-force distribution is compatible with a lognormal-like shape.

Table S3. Optimized beta and lognormal adhesion-force distribution parameters and corresponding RMSE values obtained from RnR-based inverse reconstructions of the measured resuspension curves. The RMSE values quantify the error in the fitted resuspension curves only; the corresponding adhesion-force CDFs are compared qualitatively in Figures S3 - S11.

Case	r_p [μm]	Beta			Lognormal			RMSE Difference
		p, q	F_{min}, F_{max} [nN]	RMSE	spread	reduction	RMSE	
Taheri and Bragg.[1]	10	0.7, 1.7	50, 6,000	0.0171	3.7	17	0.0395	0.0224
Ibrahim et al.[2]	16	3.5, 5	60, 1,600	0.0278	1.5	45	0.0297	0.0019
Ibrahim et al.[2]	36	1.2, 2.3	0, 1,600	0.0206	2.0	1523	0.046	0.0254
Ibrahim et al.[2]	35	0.7, 4.1	70, 1,200	0.0122	2.0	368	0.0136	0.0014
Barth et al.[3]	12.5	2.2, 2.8	42, 94	0.0192	1.2	372	0.0372	0.018
Barth et al.[3]	22	2.5, 3.7	50, 100	0.0129	1.1	598	0.0198	0.0069
Vincent et al.[4]	6	0.7, 2.3	85, 2,230	0.0094	3.3	22	0.0176	0.0082
Vincent et al.[4]	11.5	0.3, 1.3	130, 2,800	0.0218	3.7	63	0.0391	0.0173
Almog et al.[5]	4	1.3, 2.1	60, 3,400	0.0144	3.0	5	0.0198	0.0054

The main advantage of the beta formulation is not that it rejects the lognormal representation, but that it avoids imposing a specific semi-infinite distribution shape a priori. Because the beta distribution is defined on a finite interval, it can explicitly represent lower and upper adhesion-force limits. This can be useful when the measured resuspension curve contains information about the onset of resuspension, the high-removal regime, or both. By contrast, the lognormal distribution necessarily contains a positive semi-infinite upper tail.

The comparison should nevertheless be interpreted within the experimental constraints of each dataset. In cases where the measured resuspension curve does not approach complete resuspension, the high-u^* region and the upper tail of the adhesion-force CDF are not directly constrained by the data. This is relevant, for example, for datasets in which the maximum measured resuspended fraction remains below complete removal. In such cases, beta and lognormal distributions may diverge at high adhesion percentiles while remaining similar over the experimentally supported range. Therefore, the comparison supports the beta distribution as a flexible bounded reconstruction tool, rather than as a universal replacement for the lognormal distribution.

S3. Literature-based consistency checks for reconstructed adhesion forces

The inverse RnR reconstruction yields effective adhesion-force values associated with rolling detachment. These values should not be interpreted as direct pull-off adhesion forces, because the inferred force depends on the assumed rolling lever arm, commonly expressed through r_p/a, and because direct adhesion measurements often probe different mechanical pathways, surface regions, humidity conditions, and particle histories.

Nevertheless, it is useful to examine whether the reconstructed adhesion magnitudes are consistent with independently reported values for comparable particle-surface systems. Table S4 summarizes two representative glass-glass comparisons. These comparisons are intended as plausibility checks rather than strict validation of the absolute reconstructed adhesion forces.

Table S4. Literature-based consistency checks for reconstructed median adhesion forces in representative glass-glass systems.

**Dataset**	**Particle /surface**	r_p **[μm]**	r_p/a	**Reconstructed** F_{50} **[nN]**	**Reported adhesion range**	**Interpretation**
Ibrahim et al. [20]	glass/ glass	16	130, 100, 70	890, 680, 480	Biggs et al. [48]	Same order
Almog et al. [44]	glass/ glass	4	100, 70, 40	1260, 890, 510	Rush et al. [31]	Same order

These comparisons support the physical plausibility of the reconstructed adhesion magnitudes. However, because the absolute reconstructed values depend on the assumed or estimated r_p/a, the more robust outcome of the analysis is the α-normalized moment-equivalent adhesion resistance and its scaling behavior, rather than a unique direct measurement of the absolute pull-off adhesion force.
